# *Trypanosoma brucei* and *Trypanosoma cruzi* DNA Mismatch Repair Proteins Act Differently in the Response to DNA Damage Caused by Oxidative Stress

**DOI:** 10.3389/fcimb.2020.00154

**Published:** 2020-04-16

**Authors:** Viviane Grazielle-Silva, Tehseen Fatima Zeb, Richard Burchmore, Carlos Renato Machado, Richard McCulloch, Santuza M. R. Teixeira

**Affiliations:** ^1^Departamento de Bioquímica e Imunologia, Universidade Federal de Minas Gerais, Belo Horizonte, Brazil; ^2^The Wellcome Centre for Integrative Parasitology, Institute of Infection, Immunity and Inflammation, University of Glasgow, Glasgow, United Kingdom

**Keywords:** *Trypanosoma cruzi*, *Trypanosoma brucei*, DNA Mismatch Repair, MSH2, MSH6, oxidative stress

## Abstract

MSH2, associated with MSH3 or MSH6, is a central component of the eukaryotic DNA Mismatch Repair (MMR) pathway responsible for the recognition and correction of base mismatches that occur during DNA replication and recombination. Previous studies have shown that MSH2 plays an additional DNA repair role in response to oxidative damage in *Trypanosoma cruzi* and *Trypanosoma brucei*. By performing co-immunoprecipitation followed by mass spectrometry with parasites expressing tagged proteins, we confirmed that the parasites' MSH2 forms complexes with MSH3 and MSH6. To investigate the involvement of these two other MMR components in the oxidative stress response, we generated knockout mutants of MSH6 and MSH3 in *T. brucei* bloodstream forms and MSH6 mutants in *T. cruzi* epimastigotes. Differently from the phenotype observed with *T. cruzi* MSH2 knockout epimastigotes, loss of one or two alleles of *T. cruzi msh6* resulted in increased susceptibility to H_2_*O*_2_ exposure, besides impaired MMR. In contrast, *T. brucei msh6* or *msh3* null mutants displayed increased tolerance to MNNG treatment, indicating that MMR is affected, but no difference in the response to H_2_*O*_2_ treatment when compared to wild type cells. Taken together, our results suggest that, while *T. cruzi* MSH6 and MSH2 are involved with the oxidative stress response in addition to their role as components of the MMR, the DNA repair pathway that deals with oxidative stress damage operates differently in *T. brucei*.

## Introduction

Chagas disease (or American trypanosomiasis) and African Sleeping Sickness (or Human African trypanosomiasis—HAT) are two important endemic and neglected zoonoses caused by parasites of the trypanosomatidae family, respectively called *Trypanosoma cruzi* and *Trypanosoma brucei*. About 6–7 million people worldwide are estimated to be infected with *T. cruzi*. Although Chagas disease is endemic in Latin American countries, it has become a global health concern due to migration flows to Europe, United States, Canada and Japan (Antinori et al., 2017; WHO, 2019a). Besides infecting humans, *T. brucei* infects animals, including cattle, causing a form of the disease named Nagana (or African Animal Trypanosomiases-AAT) that has a major economic impact for the livestock industry in east and southern Africa (Isaac et al., 2017). Human African Trypanomiasis is caused by *Trypanosoma brucei gambiense* or *Trypanosoma brucei rhodesiense* and is frequently fatal if not treated (WHO, 2019b).

Both parasites have digenetic life cycles that involve an invertebrate host—a triatomine bug infected with *T. cruzi*, or a tsetse fly infected with *T. brucei*—and a mammalian host. Inside the digestive tract of the triatomine bug, *T. cruzi* multiplies as epimastigotes before differentiating into infective, non-replicative metacyclic trypomastigotes. After a blood meal, metacyclic trypomastigotes are expelled with the vector's feces. During a bite, eliminated parasites can enter the bloodstream when the host scratches the skin area, or through mouth mucosa, eyes and nose. Although less frequent, human infection may also happen by non-vectorial routes such as ingestion of contaminated food, blood transfusion, organ transplantation, or during pregnancy from contaminated mothers (Cevallos and Hernández, 2014; Santana et al., 2019). Circulating trypomastigotes can invade different cell types, where they replicate as intracellular replicative amastigotes that burst the cell and are released into the bloodstream with the potential to infect new cells (Brener, 1973). Similar to *T. cruzi, T. brucei* has a complex life cycle in which it has to adapt to the host bloodstream and different compartments of the tsetse fly, such as the midgut after a blood meal and then the salivary gland before transmission to a new mammalian host. Two replicative forms are most readily cultured *in vitro*: procyclic form (PCF), found in the insect vector, and bloodstream form (BSF) present in the mammalian host. Because of this experimental accessibility, most genetic analyses have focused on *T. brucei* BSF and PCF (Matthews, 2005).

To maintain their genome integrity, while adapting to survive in distinct and often hostile environments, trypanosomatids rely on various DNA repair pathways that act in response to different types of DNA damage (Machado-Silva et al., 2016). One such pathway is the DNA Mismatch Repair (MMR) pathway, which is the main pathway, widely conserved from prokaryotes to eukaryotes, that corrects replication errors that escape the proofreading activity of replicative DNA Polymerases (Li, 2008). Besides recognizing non-Watson-Crick base pairing, MMR also acts on insertion/deletion loops (IDLs), as well as on DNA damage caused by endogenous agents such reactive oxygen species (ROS) derived from cell metabolism, hydrolytic and oxidative reactions with water or exogenous sources, for example UV and ionizing radiations, alkylating agents, and crosslinking agents (Edelbrock et al., 2013). In eukaryotes, MMR initiates by the recognition of DNA mispairing by the partially redundant MSH2-MSH6 (MutSα) and MSH2-MSH3 (MutSβ) heterodimers, which are homologous to the bacterial MutS homodimer. MutSα recognizes single base pair mismatches and 1–2 base insertion/deletion loops (IDLs), while MutSβ primarily recognizes larger IDLs. When MSH2-MSH6 or MSH2-MSH3 binds to mispaired bases, a ring is formed around the DNA, with the DNA binding domain of MSH6 or MSH3 making contact with both the mispaired base and adjacent sites of the DNA. This binding results in DNA bending (Kumar et al., 2011), which works as a “double check” before DNA repair is initiated (LeBlanc et al., 2018). In addition to their DNA binding domains, MSH proteins also have an ATP binding domain. ATP activation is required for downstream events leading to DNA repair. The lesion detected by MSH proteins is repaired through enzymatic complexes that make an endonucleolytic cut on the newly synthesized strand. The ATP-activated MSH complex recruits MLH/PMS heterodimers that are homologs of bacterial MutL proteins. Together with accessory factors including PCNA, RFC, RPA, and exonuclease 1 (ExoI), MLH/PMS initiate the excision of the error-containing strand. Upon removal, the segment is re-synthesized by DNA polymerase delta and ligation by DNA ligase I restores a corrected DNA duplex (Kim et al., 2018).

Besides their primary role in MMR, eukaryotic MMR proteins are involved in diverse cellular processes such as homologous recombination (HR) (Spies and Fishel, 2015), triplet repeat expansion (Iyer et al., 2015), somatic hypermutation of immunoglobulins (Pilzecker and Jacobs, 2019) and cell signaling (Gupta and Heinen, 2019). MMR proteins activate cell cycle checkpoints and cell death pathways in response to certain DNA lesions, an additional role in the DNA damage response that can trigger cell cycle arrest and apoptosis (Li et al., 2016). Because of their importance in genome maintenance, mutations that cause loss of function in different MMR genes have been associated with predisposition to various types of cancer (Lee et al., 2016).

Genome sequence analyses revealed that *T. cruzi* and *T. brucei* possess a complete set of MMR proteins, indicating that these organisms have a functional MMR pathway composed of homologs of MSH2, MSH3, and MSH6 (originally named MSH8 in *T. brucei*) (Bell et al., 2004), MLH1 and PMS1 (Passos-Silva et al., 2010). However, no homolog for MSH1, which is the MutS homolog involved with the repair of mitochondrial DNA, including oxidative damage (Kaniak et al., 2009; Pogorzala et al., 2009; Foyer, 2018), has been identified in these organisms. We have previously investigated the role of the MSH2 and MLH1 components of the MMR pathway in *T. cruzi* and *T. brucei* by generating knockout mutants in different life cycle forms of these parasites (Bell and McCulloch, 2003; Machado-Silva et al., 2008; Campos et al., 2011; Grazielle-Silva et al., 2015). Because *T. cruzi* and *T. brucei* are exposed to several sources of oxidative stress during their life cycle and, among the different antioxidant defense mechanisms these parasites have evolved to protect genomic and mitochondrial DNA from oxidative lesions is the MMR pathway, we began investigating the role of MSH2 in response to the oxidative stress in both parasites (Machado-Silva et al., 2016). *T. brucei msh2* null mutants show increased tolerance to MNNG exposure, a phenotype characteristic of MMR impaired cells (Bell et al., 2004; Grazielle-Silva et al., 2015). In addition, in *T. brucei* bloodstream forms, the lack of TbMSH2 resulted in increased susceptibility to H_2_*O*_2_ exposure (Machado-Silva et al., 2008; Campos et al., 2011). Complementation of the TbMSH2 null mutant with *T. cruzi* MSH2 restores the oxidative stress response, but not MMR impairment as suggested by the response to MNNG treatment and microsatellite instability (MSI) assays (Machado-Silva et al., 2008). These experiments provided the first line of evidence indicating that *T. cruzi* MSH2 acts in response to the oxidative stress in a pathway or a signaling process that is independent of canonical MMR pathway. To better investigate this new role of MSH2 in response to oxidative damage in DNA, we have also tested a *T. brucei mlh1* null mutant, which showed impaired MMR, but no defect in the oxidative stress response (Bell et al., 2004; Machado-Silva et al., 2008; Grazielle-Silva et al., 2015). *T. cruzi* heterozygous *msh2* knockout mutants have increased susceptibility to oxidative stress caused by H_2_*O*_2_ and accumulate more 8-oxo-guanine in genomic and mitochondrial DNA after H_2_*O*_2_ exposure (Campos et al., 2011) but, surprisingly, deletion of the two *msh2* alleles in *T. cruzi* epimastigotes resulted in increased tolerance to oxidative damage caused by H_2_*O*_2_. Similarly, *msh2* knockout generated in *T. brucei* PCF also presented increased tolerance to H_2_*O*_2_ treatment. These results have led us to propose that an adaptation mechanism to cope with the loss of MSH2 took place in the insect stages of these mutant parasites, i.e., *T. brucei* PCF and *T. cruzi* epimastigotes, allowing them to deal with the oxidative stress in the absence of this protein (Grazielle-Silva et al., 2015). Here, we investigate the involvement of other MMR components in the oxidative stress response by analyzing the effect of various DNA damaging treatments on *T. brucei* and *T. cruzi msh3* or *msh6* knockout cell lines. Our main goal was to verify whether the previously observed response to oxidative stress involving MSH2 is part of a DNA damage response that works independently of MMR, or if other MMR proteins act together with MSH2 to repair oxidative damage in a non-canonical DNA repair pathway.

## Methodology

### Parasite Cultures

*T. brucei* cultures of the Lister 427 strain bloodstream (BSF) form were maintained at 37°C, 5% CO_2_ in HMI-9 (GIBCO) medium supplemented with 20% fetal bovine serum (GIBCO). Cell passages were performed every 48 h, with population density maintained between 1 × 10^5^ and 2 × 10^6^ cells/mL. Epimastigotes of the CL Brener clone of *T. cruzi* were maintained in logarithmic growth phase at 28°C in liver infusion tryptose (LIT) medium supplemented with 10% fetal bovine serum (GIBCO) and penicillin (10,000 U/mL)/Streptomycin (10,000 μg/mL) (GIBCO) as described (Camargo, 1964). Metacyclic trypomastigotes, obtained after metacyclogenesis of epimastigote cultures maintained in LIT medium for 15–20 days, were used to infect Vero cells cultured in high-glucose DMEM medium (GIBCO) supplemented with 5% fetal bovine serum (GIBCO) and penicillin (10,000 U/mL)/Streptomycin (10,000 μg/mL) (GIBCO).

### Plasmid Constructs to Generate Knockout and Tagged Parasites

Knockout constructs prepared to delete *msh3* (TritrypDB Tb427tmp.160.3760) or *msh6* (TritrypDB Tb427.10.6410) in *T. brucei* BSF cells have minor changes in their designs relative to those used before for *msh2* and *mlh1* knockout (Grazielle-Silva et al., 2015). The knockout constructs were generated by PCR-amplifying the 5′ and 3′ UTRs of the gene of interest from *T. brucei* Lister 427 genomic DNA. PCR amplified fragments containing the 5′ and 3′ UTRs from *msh3* and *msh6* were cloned into the knockout construct. These constructs were linearized with *Sac*I and *Xho*I and used for transfection of *T. brucei* BSF as described below. *T. brucei* BSF and PCF were also transfected with constructs to tag MSH2 and MSH6 C-terminally in their endogenous locus. Primers complementary to the C-terminal region of *msh2* CDS were used to generate an amplicon that was cloned into the plasmid pNAT12^myc^ (Alsford and Horn, 2008) resulting in the *msh2* CDS fused to 12 repeats of the c-myc epitope. Similarly, primers complementary to a C-terminal region of *msh6* CDS were designed to generate a fragment with the *msh6* CDS in frame with six repeats of the hemagglutinin (HA) epitope derived from the *T. brucei* pTbMCM-HA plasmid (Tiengwe et al., 2012).

To generate *T. cruzi* epimastigotes with the two *msh6* alleles deleted, we used a donor plasmid containing the Neomycin phosphotransferase gene (Neo) or Hygromycin B phosphotransferase gene (Hygro) flanked by intergenic sequences of the *HX1* and *GAPDH* regions derived from the pROCK_GFP vector (DaRocha et al., 2004). To generate the donor sequence named TcMSH6_HX1_Neo_GAPDH_MSH6, *Tcmsh6* coding region previously cloned in the pCR® TOPO 2.1 (Thermo Fisher Scientific) was digested with *Sac*II and *Xmn*I and ligated with T4 DNA ligase into the construct named HX1_Neo_GAPDH (Grazielle-Silva et al., 2015). The donor sequence MSH6_HX1_Neo_GAPDH_MSH6 was PCR-amplified and used together with the ribonucleoprotein (RNP) complex formed by recombinant Cas9 (rCAS9) and two sgRNAs that recognizes the 5′and 3′ portions of *Tcmsh6* CDS for transfection of *T. cruzi* CL Brener clone. To generate parasites resistant to Neomycin and Hygromycin in which the two alleles of *Tcmsh6* were deleted, another round of transfection was performed using the RNP complex described above and the donor sequence named MSH6_HX1_Hygro_GAPDH_MSH6. The donor sequence with the Hygro gene was constructed after digesting the Topo_HX1_Hygro_GAPDH plasmid, in which the insert was cloned in the reverse orientation, with *Kpn*I and *EcoR*V. The fragment of interest was cloned in the pGEM_TcMSH6 vector previously digested with *Cla*I and *Kpn*I. This construction was designed in a way that HX1_Hygro_GAPDH disrupts *Tcmsh6* CDS.

To generate *T. cruzi msh6* knockouts complemented with *msh6*, two knockout clones (cl1 and cl2) were transfected with the pTREX plasmid (Vazquez and Levin, 1999) containing the MSH6 fused to an HA tag and the puromycin resistance gene. A PCR fragment with the *msh6* CDS and six in frame repeats of the HA epitope and the restriction enzymes *Spe*I and *Xho*I was cloned into the pTREX_GFP-PAC plasmid digested with *Xba*I and *Xho*I. Three days after transfection the parasite populations growing in medium with 15 μg/mL of puromycin were tested by Western blot and treatments with MNNG and H_2_*O*_2_.

To evaluate the cellular localization of MSH6, *T. cruzi* epimastigotes were transfected with a modified version of pTREX plasmid (Vazquez and Levin, 1999) to express MSH6 fused to the monomeric red fluorescent protein (mRFP). The *msh6* CDS without the stop codon was amplified with primers that also added the restriction sites for *Xba*I and *Sma*I to the PCR fragment. After digestion, this fragment was cloned into pTREX_mRFP giving origin to pTREX_MSH6_mRFP. To C-terminally tag MSH6 at its endogenous locus, primers were generated against a C-terminal region of *msh6* CDS excluding the stop codon. This fragment was cloned into pCR® TOPO 2.1 (Thermo Fisher Scientific) in frame with the six repeats of the HA epitope derived from the *T. brucei* pTbMCM-HA plasmid (Tiengwe et al., 2012). As a selective marker, neomycin resistance gene derived from the pROCK_Neo plasmid (DaRocha et al., 2004) was also cloned in the same pCR® TOPO 2.1 (Thermo Fisher Scientific) vector. The construct was linearized with *Kpn*I and used to transfect *T. cruzi* epimastigotes.

Primers used to generate knockout constructions, plasmids to express tagged proteins and to evaluate the correct insertion of knockout constructions in the genome are listed in Supplementary Table 1.

### *T. brucei* Transfection

Transfection of *T. brucei* BSF was performed using the AMAXA Nucleofactor kit (Amaxa Biosystems) and the X-001 programme. Cultures were grown to a density of 1 × 10^6^ cells/mL and around 4 × 10^7^ cells were used per transfection with 5–10 μg of linearized DNA, as previously described (Burkard et al., 2007).

### *T. cruzi* Transfection With Recombinant Cas9

Transfection to generate T. cruzi knockout cells using the CRISPR/Cas9 system were performed as previously described (Burle-Caldas et al., 2018). Briefly, Cas9/sgRNAs RNP complex plus a donor sequence composed of Tcmsh6 CDS replaced by the resistance gene construction HX1_Neo_GAPDH (named MSH6_HX1_Neo_GAPDH_MSH6) were used to transfect T. cruzi epimastigotes from the CL Brener cloned strain. Recombinant Cas9 derived from Staphylococcus aureus and two sgRNAs that were transcribed *in vitro* formed the ribonucleoprotein (RNP) complex that was transfected together with the donor cassette with the Tb-BSF buffer (Schumann Burkard et al., 2011) and the Amaxa Nucleofector (Lonza) program U-033. After one pulse, parasites were cultivated in LIT medium with 200 μM G418 (GIBCO). After selection of G418 resistant parasites, another round of transfection was performed as described above using the donor sequence MSH6_HX1_Hygro_GAPDH_MSH6 and selection with 200 μM Hygromicin B (Thermo Fisher).

### Cellular Localization

*T. cruzi* expressing MSH6 fused to mRFP or to 6x HA epitopes were fixed with 4% paraformaldehyde for 5 min, permeabilized with 0.1% Triton X-100 for 10 min, blocked with 1% BSA, 0.2% Tween 20 for 1 h at room temperature and incubated with 1:500 anti-HA antiserum (Roche) for 1 h. After washing with PBS, nuclei were stained with 1 μg/mL of DAPI (Molecular Probes/ Life Technologies) for 5 min and cover slides mounted with prolong gold anti-fade solution (Molecular Probes/Life Technologies). Images were acquired with a 100x objective in the fluorescence microscope Olympus BX60 microscope using Q-color 5 digital camera and Qcapture Pro 6.0 software and with Nikon Eclipse Ti Tecnai G2-12 SpiritBiotwin FEI (120kV) at the Centro de Aquisição e Processamento de Imagens (CAPI-ICB/UFMG).

### Immunoprecipitation of MMR Components

Pulldown of TbMSH2 with c-myc tag was performed as described (Tiengwe et al., 2012). Briefly, 10^9^ cells of *T. brucei* BSF or PCF co-expressing TbMSH2 fused to c-myc tag (TbMSH2::myc) and MSH6 fused to HA tag (MSH6::HA) were lysed in lysis buffer (50 mM Hepes [pH7.55]; 100 mM NaCl; 1 mM EDTA [pH8.0]; 10% glycerol; 1% Triton X-100) for 2 h at 4°C and incubated with anti-myc antibody (Milipore) previously bound to Dynabeads M-280 Sheep Anti-Mouse IgG (ThermoFisher) for 2 h at 4°C. After five washing steps with wash buffer (50 mM Hepes [pH7.55]; 100 mM LiCl; 1 mM EDTA [pH8.0]; 1 mM EGTA; 0,7% Na Deoxycolate; 1% NP-40) and one step washing with TE wash buffer (10 mM Tris [pH8.0], 1 mM EDTA [pH8.0]; 50 mM NaCl), beads were collected using a magnetic rack and bound protein with its binding partners released in elution buffer (50 mM Tris [pH8.0], 10 mM EDTA [pH8.0]; 1% SDS). Samples (input and pulldown) were analyzed by SDS-polyacrylamide gel electrophoresis and Western blotting with anti-myc antibody against the pulled down TbMSH2 and with anti-HA antibody against TbMSH6.

*T. cruzi* immunoprecipitation was performed after lysing epimastigotes WT and transfected parasites co-expressing MSH6 fused to HA tag (MSH6::HA) and MSH2 fused to c-myc tag (MSH2::myc) in RIPA buffer (0 5 mM Tris-HCl pH 8.; 15 mM NaCl; 0,1%NP-40, 0.05% Deoxicolato de sódio, 0.01% SDS). After lysis, cell extracts were centrifuged 8,2,000 × g for 10 min and the supernatant incubated with EZview Red Anti-HA Affinity Gel (Sigma—Aldrich) at 4°C under soft agitation for 16 h. Ten percentage of cell lysate (input fraction) were transferred to a new tube and saved for further western blot analysis. After incubation period beads were centrifuged 8,2,000 × g for 1 min and unbound proteins, present in the supernatant, transferred to new tube. Beads were washed five times with lysis buffer and bound proteins eluted in elution buffer (62,5 mM Tris-HCl pH 6,8; 10% glycerol; 2% SDS; 5% β-mercaptoethanol; 0,002% bromophenol blue). Samples were analyzed by SDS-polyacrylamide gel electrophoresis and Western blotting with anti-HA antibody against the pulled down TcMSH6 and with anti-myc antibody against TcMSH2.

### Parasite Treatment With Genotoxic Agents

*T. brucei* BSF cultures were inoculated in HMI-9 medium (Hirumi and Hirumi, 1994) at density of 1 × 10^5^ cells/mL. The conversion of Alamar blue to fluorescent Resazurin (Räz et al., 1997) was used to determine the sensitivity of *T. brucei* WT cells and *Tbmsh3* and *Tbmsh6* mutants toward MNNG. Briefly, 200 μl of 1 × 10^5^cells/mL cultures were plated on polystyrene 96 well-plates in the presence of medium containing doubling dilutions of MNNG from 400 to 0.39 μM. After 48 h, 20 μl of Resazurin (0.125 mg/mL) was added to each well. Cultures were allowed to grow for another 24 h and then florescence was measured using luminescence spectrometer (LS 55, Perkin Elmer) at an emission wavelength of 590 nm. IC50 values was calculated using Prism (GraphPad). For treatment with H_2_*O*_2_ BSF cells were diluted to 1 × 10^6^ cells/mL in HMI-9 medium and incubated with 100 μM H_2_*O*_2_ (VWR) at 37°C, 5% CO_2_ for 72 h. After growth, cell density was measured using a haematocytometer.

WT *T. cruzi* epimastigotes and *msh6* mutants in the exponential growth phase were counted and diluted to 1 × 10^7^ cells/mL in LIT medium in the absence or presence of 5 μM MNNG (Tokyo chemical industry Ltd). After 72 h cell densities were determined by counting live cells with a haematocytometer using Erythrosin B exclusion and plotted as the percentage survival of the MNNG treated cells relative to untreated cultures. For treatment with H_2_*O*_2_, *T. cruzi* epimastigotes were incubated in 100 μM H_2_*O*_2_ for 30 min in PBS 1x, after which H_2_*O*_2_ containing PBS was removed and cells allowed to grow in LIT medium for 72 h. Cell viability was determined with a haematocytometer using Erythrosin B exclusion and plotted as percentage survival of the treated cells relative to untreated. Similar to described above, *T. cruzi msh6* knockouts transfected with pTREX_MSH6::HA_PAC were treated with 5 μM MNNG and 100 μM H_2_*O*_2_. 72 h after drug incubation cell numbers were determined with a haematocytometer using Erythrosin B exclusion and plotted as the percentage survival.

### DNA Sequencing

Two different clones of *T. cruzi msh6* CDS amplified by PCR and cloned in the pCR® TOPO 2.1 (Thermo Fisher Scientific) were selected for DNA sequencing by capillary electrophoresis using an ABI Prism 3730 Genetic Analyzer (Applied Biosystems). Four different primers were used to cover the complete *msh6* sequence.

### Cell Infection

Vero cells were infected with *T. cruzi* metacyclic trypomastigotes and maintained at 37 °C in a humidified atmosphere containing 5% CO_2_ until trypomastigotes were released in the supernatant. These trypomastigotes were counted using a haemocytometer and an equal number of WT and two *msh6* null mutant clones were used to infect Vero cells or macrophages harvest from Balb/C mice after thioglycollate injection, previously adhered to 13 mm glass coverslips inside 24 wells-plates. At a MOI of 5 parasites per cell, trypomastigotes were allowed to infect cells for 2 h after which non-internalized trypomastigotes were removed. Time points 24 and 48 h after infection were fixed and stained with panoptic. Five hundred uninfected and infected cells counted in random fields. Intracellular amastigotes of infected cells were also counted. The data was represented by the infection index = percentage of infected cells × mean number of parasites per infected cells.

### Statistical Analyses

Statistical analyses in this work were performed using the GraphPad Prism version 7.00 for Mac OS X, GraphPad Software, La Jolla California USA, www.graphpad.com. Data are presented as mean plus standard deviation, and all experiments were repeated at least three times. Results were analyzed for significant differences using ANOVA followed by Bonferroni post-test. Statistical tests used are described at each figure legend. The level of significance was set at *P* < 0.05.

## Results

### MSH2 Forms Heterodimers With MSH3 and MSH6

**Figure 1:**
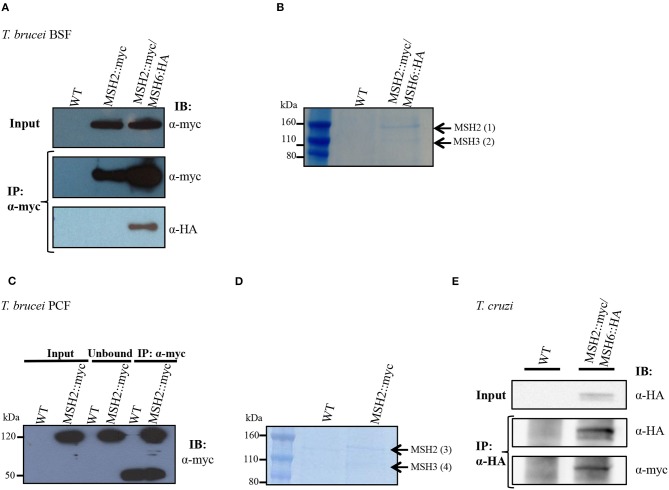
Interaction of MSH2 with MSH6 and MSH3 in *T. brucei* and *T. cruzi*. **(A)** Cell extract of *T. brucei* BSF wild type (WT), BSF expressing MSH2 with a myc tag (MSH2::myc) and BSFs co-expressing MSH2::myc and MSH6 with an HA tag (MSH6::HA) were used for co-immunoprecipitation with α-myc antibody bound to magnetic beads. Western blot of input and precipitated (IP) fractions were incubated with α-myc (1:7,000) or α-HA (1:5,000) antibodies. **(B)** Polyacrylamide gel electrophoresis stained with Brilliant Blue G showing protein bands present on the IP fraction described in Figure 1A. The bands identified in MSH2:myc/MSH6::HA expressing parasites but not on WT BSFs were cropped and sent for mass spectrometry. Black arrows show identified MSH2 and MSH3 proteins. **(C)** Cell extract of *T. brucei* PCF WT and PCF expressing MSH2::myc were used to immunoprecipitate MSH2 with α-myc. The IP fraction as well as the unbound and input fractions were analyzed on western blot with anti-myc antibodies. In addition to MSH2, a 50 KDa band corresponding to immunoglobulin heavy chain is identified in the IP fraction. **(D)** IP fractions from WT and PCF expressing MSH2::myc were separated on polyacrylamide gel electrophoresis stained with Brilliant Blue G. The two bands present in the IP fraction of MSH2::myc expressing cells but not on WT PCFs were cropped and sent to mass spectrometry. Arrows show identified MSH2 and MSH3 proteins. **(E)** Cell extract of *T. cruzi* epimastigote WT and epimastigotes transfected to co-express MSH2::myc and MSH6 with an HA tag (MSH6::HA) were used for co-immunoprecipitation with α-HA antibody. Western blot of input and precipitated (IP) fractions were incubated with α-HA (1:3,000) or α-myc (1:3,000) antibodies.

Previous studies have shown that *T. cruzi* and *T. brucei* MSH2 are involved in the oxidative stress response to DNA damage (Grazielle-Silva et al., 2015). To investigate the mechanisms behind this additional role of MSH2 we searched for proteins that may interact with MSH2 by tagging *T. brucei* MSH2 with the myc epitope and performing immunoprecipitation. As shown in Figure 1A, *T. brucei* MSH2::myc forms a complex with MSH6 in BSFs co-expressing HA-tagged MSH6. Protein bands identified after SDS-PAGE of the immunoprecipitated fraction obtained from cells co-expressing MSH2::myc and MSH6::HA, but not from WT BSFs, were sent for protein sequencing and identification using mass spectrometry. As shown in Figure 1B, the two major bands present in the immunoprecipitated pellet were identified as MSH2 and MSH3. Similarly, PCFs expressing MSH2 with the myc epitope were used for immunoprecipitation with anti-myc antibody as shown on western blot in Figure 1C. Mass spectrometry analyses of the proteins present in the two bands shown in Figure 1D showed that, in transfected PCF parasites, but not in WT cells, MSH3 co-immunoprecipitated with myc-tagged MSH2 (Figure 1D). Supplementary Table 2 shows identified proteins and peptide sequences obtained after mass spectrometry. Similar to the data obtained in *T. brucei*, immunoprecipitation of cell extracts from *T. cruzi* epimastigotes co-expressing MSH6::HA and MSH2::myc showed the presence of the MSH2-MSH6 complex (Figure 1E). In conclusion, these analyses showed that MSH2 forms a complex with both MSH3 and MSH6 in *T. brucei* BSFs. In *T. brucei* PCFs we identified a complex containing MSH2 and MSH3, and in *T. cruzi* epimastigotes MSH2 was found to interact with MSH6.

### *T. brucei msh6* and *msh3* Null Mutants Present a Similar Response to Oxidative Stress When Compared to Wild Type Parasites

**Figure 2:**
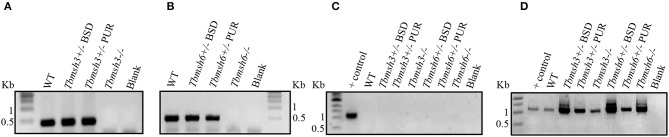
RT-PCR analysis of BSF *msh3* and *msh6* mutants. Agarose gel electrophoresis of PCR products generated after reverse transcriptase (RT) reactions of RNA isolated from *T. brucei* BSFs *msh3* and *msh6* knockout mutants (-/-), WT cells and heterozygous (+/-) mutants. **(A)** PCR products generated with cDNA from WT and *Tbmsh3* mutants and primers that amplify part of *msh3* CDS with an expected fragment of 437 bp. **(B)** PCR with cDNA from *Tbmsh6* mutants and primers that amplify part of *msh6* CDS with an expected fragment of 502 bp. **(C)** PCR with RNA samples in the absence of RT isolated from wild type and mutants and *TbRAD51* primers; gDNA from WT *T. brucei* was used as a positive control for the reaction, and blank is a control PCR with water instead of a DNA template. PCR-amplification has the expected size 1,122 bp. **(D)** PCR products from cDNA samples derived from RNA isolated from samples as shown in **(C)** and amplified with *Tbrad51* primers. PCR-amplification has the expected size 1,122 bp.

The first step during DNA error recognition by the eukaryotic MMR is performed by heterodimers formed between MSH2 and MSH6, named MutSα, or MSH2 and MSH3, named MutSβ. We showed that both complexes are formed in *T. brucei* and, as described previously, that MSH2 is involved in the response to oxidative damage in DNA. Since we have also evidences indicating that MSH2 works independently from downstream effector proteins, like MLH1, in the response to oxidative damage (Grazielle-Silva et al., 2015), it is possible that such activity is part of a non-canonical MMR pathway. To test the possibility that MSH2 is the only protein of the MMR pathway responsible for the recognition of oxidative damage, we generated *T. brucei* BSF and PCF mutants with deleted *msh3* or *msh6*. *Tbmsh6* and *Tbmsh3* knockouts were obtained by disrupting their coding sequences (CDS) with constructs containing blasticidin or puromycin drug resistance genes flanked by tubulin and actin intergenic regions to provide SL addition and poly-A processing signals. After transfection, clones resistant to blasticidin and puromycin were tested by PCR to confirm gene disruption (Supplementary Figure 1). As shown in Figure 2, PCR analyses of cDNAs generated from RNA isolated from WT and drug resistant parasites using primers that bind specifically to the *Tbmsh3* or *Tbmsh6* CDSs showed no expression of *msh3* or *msh6* mRNAs in knockout parasites (Figures 2A,B). PCR reactions performed with RNA samples that were not previously incubated with Reverse Transcriptase generated no products, except with control DNA (Figure 2C). To attest the quality of the cDNAs produced from all samples, the *Tbrad51* CDS was amplified with specific primers, generating the expected PCR products (Figure 2D).

**Figure 3:**
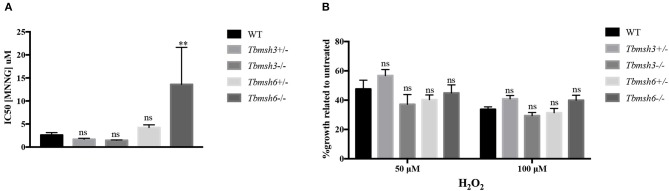
Evaluation of MMR deficiency in *T. brucei msh3* and *msh6* knockouts. **(A)** The conversion of Alamar blue to fluorescent Resazurin was used to determine the sensitivity of *T. brucei* WT cells and *Tbmsh3* and *Tbmsh6* mutants toward MNNG and shown by IC50 values. IC50 values are the mean of three experiments. **(B)** Susceptibility of *T. brucei* MMR knockout mutants to the oxidative stress caused by H_2_*O*_2_. WT, *Tbmsh3*+/-, *Tbmsh3*-/-, *Tbmsh6*+/-, and *Tbmsh6*-/- were grown in culture media containing 0, 50, or 100 μM H_2_*O*_2_. Cell densities were counted 72 h after incubation beginning. Values are shown as percentage survival, which was calculated from the cell density of each cell type in the presence of H_2_*O*_2_ as a percentage of the same cells grown in the absence of damage (which was taken as 100%). Vertical lines show standard deviation. **p < 0.01: determined by one-way ANOVA with Bonferroni post-test of knockout mutants relative to WT cells. Ns indicates no significant difference.

A phenotype frequently associated with MMR impairment is increased tolerance to MNNG exposure (de Wind et al., 1995). Using the alamar blue assay to determine cell viability after treatment of WT and *T. brucei* knockout mutants with MNNG, we showed that, while *Tbmsh6* null mutants are more resistant to MNNG exposure compared to WT cells, *Tbmsh3* null mutants have no alteration regarding tolerance to MNNG (Figure 3A).

To verify whether *Tbmsh3* or *Tbmsh6* null mutants respond differently to oxidative stress, *T. brucei* WT BSF and *Tbmsh6* or *Tbmsh3* mutants were grown in culture media containing 0, 50, or 100 μM H_2_*O*_2_ and parasite survival determined 72 h later. As shown in Figure 3B, distinct from the results previously described with *Tbmsh2* null mutants (Machado-Silva et al., 2008; Grazielle-Silva et al., 2015), no changes in the susceptibility to H_2_*O*_2_ of *Tbmsh3* or *Tbmsh6* knockout cells were observed when compared to WT parasites.

### *T. cruzi MSH6*, Encoded by a Heterozygous Single-Copy Gene, Localizes to the Nucleus

**Figure 4:**
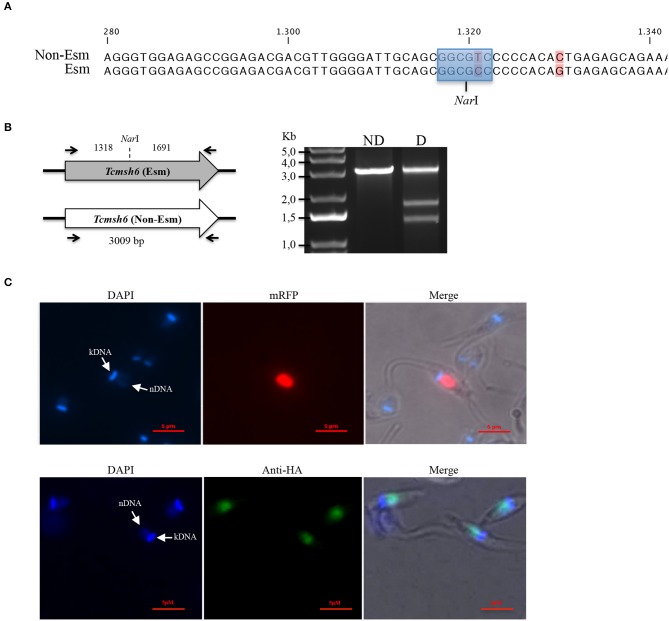
Characterization of *T. cruzi* MSH6. **(A)** A recognition site for the restriction enzyme *Nar*I (highlighted in blue) is present in the DNA sequence for the Esmeraldo-like allele but is absent in the Non-Esmeraldo-like allele of the *msh6* gene in the CL Brener clone. Non-conserved nucleotides are shown in pink. **(B)** Left panel shows that PCR amplified CDS of the Esmeraldo-like allele after digestion with *Nar*I, resulting in DNA fragments of 1,318 bp and 1,691 bp, while the non-digested fragment corresponding to *Tcmsh6* CDS has 3,009 bp. Black arrows denote primer binding site. Agarose gel electrophoresis of the PCR product from amplified Tc*msh6* CDS not digested (ND) and digested (D) with *Nar*I. **(C)** Epimastigotes transfected with the pTREX_MSH6::mRFP were analyzed under fluorescence microscopy. Parasite nucleus and kinetoplast DNA are stained with DAPI (upper panel). Parasites transfected with a plasmid that inserts an HA tag in the *msh6* locus were analyzed by immunofluoresce after stained with anti-HA antibody (1:50) and anti-rat conjugated with Alexa 488 (1:100). Parasite nucleus and kinetoplast DNA are stained with DAPI (lower panel). (Bar = 5 μM).

Analyses of the *T. cruzi* and *T. brucei* genomes have shown that *msh6* is present as a single-copy gene in both parasites (Berriman et al., 2005; El-Sayed et al., 2005). Because CL Brener, the *T. cruzi* reference strain, is a hybrid strain, most genes have been assigned to one of the two haplotypes, named Esmeraldo and Non-Esmeraldo, that compose the CL Brener genome (El-Sayed et al., 2005). When we searched for *msh6* in the CL Brener genome database (available at https://tritrypdb.org) we identified only one sequence annotated as belonging to the non-Esmeraldo haplotype. However, during our initial attempts to generate a *Tcmsh6* null mutant using donor sequences with homology to *Tcmsh6* flanking a drug resistance marker, we observed that only clones with a single deleted allele were generated. This result prompted us to investigate whether the CL Brener genome contains two alleles instead, with sequences that are different enough to prevent homologous recombination with the same donor sequence. To test this hypothesis, we amplified the *msh6* locus using primers annealing at the beginning and the end of *msh6* CDS and cloned the PCR product into pCR® TOPO 2.1 (Thermo Fisher Scientific). By sequencing plasmid DNA from different clones, we verified that the DNA sequence for *Tcmsh6* currently annotated in the *T. cruzi* CL Brener genome database represents a hybrid between the Esmeraldo-like and Non-Esmeraldo-like alleles (Supplementary Figure 2A), a frequent error that occurred during the assembly process (Daniella Bartholomeu, personal communication). Based on the PCR sequences, we concluded that the *Tcmsh6* has two alleles that present 94.2% homology at the nucleotide level and the translated product generates two distinct protein isoforms presenting 96.9% amino acid identity (Supplementary Figure 2B). To confirm the results from our sequence analyses, we digested the PCR products corresponding to *Tcmsh6* CDSs with the enzyme *Nar*I. As shown in the upper panel of Figure 4A, according to its sequence, the Esmeraldo-like *msh6* allele has the *Nar*I restriction site whereas the non-Esmeraldo allele cannot be digested by *Nar*I. Agarose gel electrophoresis of the digested products showed two fragments with 1,318 and 1,691 bp corresponding to the Esmeraldo-like allele, and one fragment with the expected size (3,009 bp) of the non-digested Non-Esmeraldo-like allele (Figure 4B). Thus, contrary to the data currently available in the CL Brener genome database, this *T. cruzi* strain contains two distinct *msh6* alleles, which can be distinguished by the presence/absence of a *Nar*I restriction site.

By expressing TcMSH6 ectopically as a C-terminal fusion with monomeric Red Fluorescence Protein (mRFP), we were able to verify that, similar to *T. cruzi* and *T. brucei* MSH2 (Grazielle-Silva et al., 2015), TcMSH6 has a nuclear localization (Figure 4C, upper panel**)**. We confirmed the MSH6 nuclear localization by generating *T. cruzi* epimastigotes expressing the protein with an HA tag inserted in the endogenous *msh6* locus (Figure 4C, lower panel). Using western blot, we showed that TcMSH6 fused to an HA tag is expressed in epimastigotes as a protein with the expected molecular weight of 117.4 kDa (Supplementary Figure 3). Thus, identical nuclear localization of TcMSH6 was observed using two different approaches, i.e., by ectopically expressing the protein fused to mRFP using an episomal vector or by expressing a HA-tagged protein inserted in its endogenous genomic locus.

### *T. cruzi msh6* Null Mutants Display Impaired MMR and Increased Susceptibility to Hydrogen Peroxide

**Figure 5:**
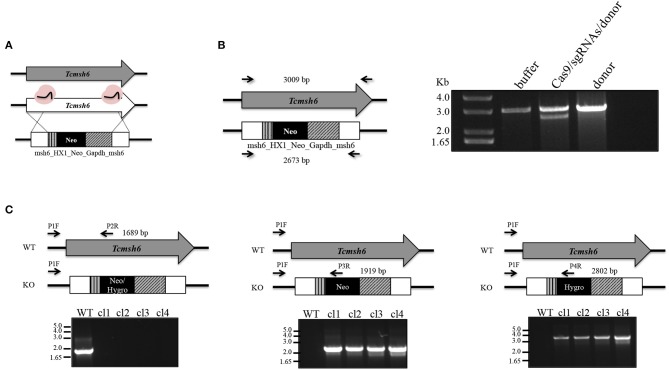
Generation of *T. cruzi msh6* null mutants. **(A)** Schematic representation of the strategy to generate *Tcmsh6* knockouts. *T. cruzi* CL Brener WT parasites were transfected with buffer, recombinant rSaCas9 (in pink), two different sgRNA sequences and a donor construction named msh6_HX1_Neo_Gapdh_msh6 or donor sequence only. **(B)** After transfection, genomic DNA was extracted and PCR-amplified using primers to amplify the entire CDS (black arrows mark primer binding site—left panel). Agarose gel electrophoresis of PCR fragments to evaluate knockout efficiency. Non-interrupted *Tcmsh6* CDS results in a PCR fragment of 3,009 bp. CDS interrupted by donor construction results in a PCR fragment of 2,673 bp. **(C)** Assessing the integration of DNA constructions to delete *Tcmsh6*. The panel above each agarose gel represents only one allele of WT or knockout (KO) culture. Black arrows denote regions of the WT or mutated loci complementary to the different primers. PCR amplifications were carried out to verify the knockout of *Tcmsh6* using specific primers annealing in the target gene (P1F and P2R) or in the Neo (P3R) or Hygro (P4R) resistance genes. PCR products generated using genomic DNA from *T. cruzi* WT or four KO clones in which Neomycin resistance gene and Hygromycin resistance have integrated are shown after separation on 1% agarose gels.

To initiate the mismatch repair mechanism, MSH2 must form a heterodimer with MSH6 or MSH3. To verify if *T. cruzi* MSH2 works as a dimer with MSH6 in response to the oxidative stress, we generated *msh6* knockout cell lines using the CRISPR/Cas9 protocol. Recently, a *T. cruzi* gene editing protocol using recombinant Cas9 derived from *Staphylococcus aureus* (rSaCas9) expressed in *Escherichia coli* was described (Soares Medeiros et al., 2017; Burle-Caldas et al., 2018). We transfected epimastigotes with two sgRNAs complexed with rSaCas9 together and a donor sequence fragment. As shown in Figure 5A, the donor fragment contains *msh6* sequences and the Neomycin phosphotransferase gene (Neo^R^) flanked by intergenic sequences that provide trans-splicing and poly-A addition sites (HX1_Neo_Gapdh cassette), as well as sequences corresponding to 5′ and 3′ regions of the *msh6* gene. After transfection DNA from G418 resistant parasites was verified by PCR analyses. While *Tcmsh6* coding sequence has 3,009 base pairs (bp), when the *msh6* CDS is replaced by the Neo^R^ cassette, the PCR product using the same primer pair generated a smaller fragment with 2,673 bp (Figure 5B). Figure 5B also showed that without G418 drug selection, in the absence of the Cas9 RNP, no parasites with disrupted *msh6* gene were generated. After transfection with the Cas9 RNP and donor DNA, only clones with one allele disrupted were generated. To disrupt the second allele, we performed a second round of transfection of one heterozygous mutant cloned cell line that is G418 resistant. PCR analyses of DNA extracted from four double resistant cloned cell lines showed that the 3.009 pb fragment was replaced by the Hygromycin cassette, which has 3.520 bp and contains the hygromycin resistance gene flanked by trans-splicing and poly-A addition signals (HX1_Hygro_Gapdh cassette) as well as 5′ and 3′ *msh6* sequences. As shown in Figure 5C the correct insertion of the Neo^R^ and Hyg^R^ cassettes, interrupting both *msh6* alleles, was confirmed by PCR-amplification with primers annealing in the *msh6* 5′ UTR and CDS (P1F—P2R, see left panel), in the *msh6* 5′ UTR and in the Neo^R^ gene (P3R or P4R, see middle panel) and in the *msh6* 5′ UTR and the HygR gene (P1F and P2R, right panel). The PCR-amplified *msh6* fragment of 1,689 bp was observed only in WT parasites but not in the four *Tcmsh6* null mutants cell lines. On the other hand, PCR fragments amplified with the *msh6* and NeoR primer of 1.919 bp and with the *msh6* and HygroR of 2,802 bp were only detected with DNA from transfected cloned cell lines and not with DNA from WT parasites (Figure 5C).

**Figure 6:**
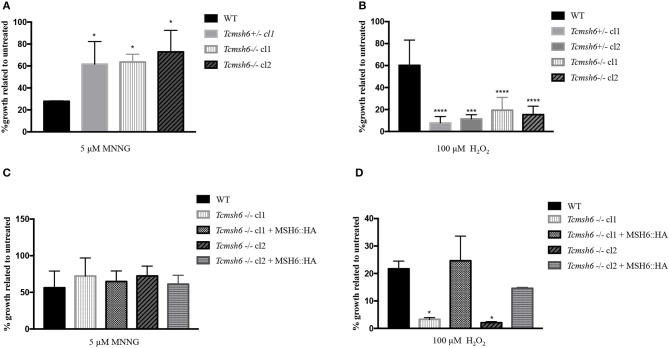
Evaluation of susceptibility of *T. cruzi msh6* mutants to N-methyl-N'-nitro-N- nitrosoguanidine (MNNG) and H_2_*O*_2_. **(A)** *T. cruzi* WT and MSH6 mutants (*Tcmsh6*+*/-* and *Tcmsh6-/-*) were grown in culture medium with 0 μM or 5 μM MNNG. Cell viability was measured after 72 h and is plotted as the percentage survival of the MNNG treated cells relative to untreated cultures. Vertical lines indicate standard deviation. The graph represents the average of two independent experiments performed in duplicate. **(B)** *T. cruzi* epimastigote cells wild type (WT), *Tcmsh6*+/- (clones 1 and 2) and *Tcmsh6-/-* (clones 1 and 2) mutants were incubated in the presence or absence of H_2_*O*_2_ 100 μM for 30 min in PBS 1x and then allowed to grow in LIT medium for 72 h, after which cell viability was determined and plotted as percentage survival of the treated cells relative to untreated. **(C)** *T. cruzi* WT, *Tcmsh6-/-* and *Tcmsh6-/-* transfected to express MSH6::HA were grown in culture medium with 0 μM or 5 μM MNNG. Cell viability was measured after 72 h and is plotted as the percentage survival of the MNNG treated cells relative to untreated cultures. Vertical lines indicate standard deviation. The graph represents the average of two independent experiments, each performed in duplicate. **(D)** *T. cruzi* WT, *Tcmsh6-/-* and *Tcmsh6-/-* transfected to express MSH6::HA mutants were incubated in the presence or absence of 100 μM H_2_*O*_2_ for 30 min in PBS 1x and then allowed to grow in LIT medium for 72 h, after which cell viability was determined and plotted as percentage survival of the treated cells relative to untreated. Data represent the average of three independent experiments, each performed in duplicate. Vertical lines show standard deviation. ^****^p < 0.0001, ^***^p < 0.001, ^*^p < 0.1: determined by one-way ANOVA with Bonferroni post-test of mutants relative to wild type cells.

To evaluate if TcMSH6 knockout affects the MMR capacity of the mutant cell lines, parasites were treated with MNNG and compared with treated WT cells. As shown in Figure 6A, a significant increase in the tolerance to MNNG treatment was observed in all *msh6* mutants, including the *Tcmsh6* heterozygous (*Tcmsh6*+*/-*) and null mutants (*Tcmsh6-/-*), compared to WT parasites. To ask if the phenotype observed with *Tcmsh2* null mutants in response to treatment with H_2_*O*_2_ is also observed in *Tcmsh6* mutants, we compared the growth of WT cells and two cloned heterozygous mutants, as well as two cloned homozygous *msh6* mutants, after treatment for 30 min with 100 μM H_2_*O*_2_ and incubating in LIT medium for 72 h. As shown in Figure 6B *Tcmsh6*+*/-* and *Tcmsh6-/-* mutants are more susceptible to the treatment with H_2_*O*_2_ than WT cells. To analyze the reversion of the *msh6* knockout phenotype, we transfected two *Tcmsh6-/-* cloned cell lines with the pTREX vector containing the complete *msh6* coding region fused to an HA tag. Western blot analyses with anti-HA antibody showed the expression of MSH6::HA in cell extracts derived from transfected cultures (Supplementary Figure 4). Although no significant differences in the tolerance to MNNG were observed, both MSH6 re-expresser cell lines showed reversion of the susceptibility to H_2_*O*_2_ treatment (Figures 6C, D).

**Figure 7:**
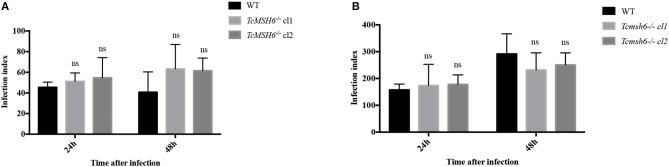
Assessment of *T. cruzi msh6* null mutants infectivity *in vitro*. **(A)** *T. cruzi* trypomastigote cells released by Vero cells infected with either WT or with two cloned cell lines of *Tcmsh6-/-* mutants were counted and equal numbers were used to infect Vero cells attached to glass coverslips. **(B)** Infection of intraperitoneal macrophages extracted from Balb/C mice with trypomastigotes released from infected Vero cells. Twenty-four and forty-eight hours post infection the infection index was estimated. Infection index = percentage of infected cells × mean number of parasites per infected cells. Values are expressed as means ± SD of one representative experiment performed in triplicate. Ns indicates no significant difference: two-way ANOVA with Bonferroni post-test of knockout mutants relative to wild type.

Because we observed that *msh6* mutants are more sensitive to H_2_*O*_2_ treatment, we investigated whether deletion of *msh6* gene affects parasite intracellular survival and amastigote multiplication. Different from *T. brucei, T. cruzi* has a part of its life cycle as an intracellular amastigote stage, which must cope with the generation of ROS by infected cells (Paiva et al., 2018). We investigated the role of MSH6 in response to the oxidative stress generated by two different cell types: epithelial cells and intraperitoneal macrophages extracted from BALB/c mice. Both cell types, previously attached to a glass coverslip, were infected with equal numbers of WT and *Tcmsh6-/-* trypomastigotes released from infected Vero cells. As shown in Figure 7 no differences in the infection index were observed between the WT and *Tcmsh6-/-* after infection of Vero cells (Figure 7A) or macrophages (Figure 7B). Supplementary Figure 5 shows that, for infected Vero cells, we observed no differences in the number of infected cells (Supplementary Figure 5A) or in the number of intracellular amastigotes per infected cells at 24 h or 48 h post-infection (Supplementary Figure 5B). Similarly, no differences in the number of infected macrophages (Supplementary Figure 5C), or in the number of intracellular parasites per infected macrophages (Supplementary Figure 5D), were observed between WT and *Tcmsh6-/-* mutants.

## Discussion

We have shown previously that the response to oxidative DNA damage works differently in *T. brucei* and *T. cruzi*, and that this response is dependent on the life cycle stage of each parasite. A summary of the responses, to the two genotoxic agents, observed in the different *T. brucei* and *T. cruzi* knockout mutants is provided in Supplementary Table 3. In *T. brucei*, MSH2 appears to be directly involved with the response to H_2_*O*_2_ exposure, since loss of MSH2 in BSFs resulted in increased sensitivity to the oxidative agent, whereas MSH2 loss in PCF cells resulted in increased tolerance to H_2_*O*_2_. Increased tolerance to H_2_*O*_2_ exposure was also seen in *T. cruzi* epimastigote MSH2 null mutants, which also showed increased survival in ROS-producing macrophages, compared with WT parasites. Similar to the phenotype observed in other eukaryotes, loss of MSH2 resulted in impaired MMR, both in *T. brucei* and *T. cruzi* mutants, as demonstrated by the increased tolerance to the alkylator MNNG. Because altered H_2_*O*_2_ tolerance was not observed in *T. brucei* BSF or PCF cells lacking MLH1, a protein that acts downstream from MSH2 in the MMR pathway, we hypothesized that MSH2 displays a dual role, one as an MMR component and a second role as a factor involved in the response to oxidative stress, in a way that is independent of MLH1. We have also hypothesized that the increased oxidative resistance of *msh2* null mutants generated in procyclic *T. brucei* and in *T. cruzi* epimastigotes may be due to adaptation of the insect stages of these parasites to MSH2 loss. This hypothesis is corroborated by the necessity of having a gradual process to obtain *T. cruzi* epimastigotes mutants with both *msh2* alleles deleted. We have also speculated that the differences observed in the phenotypic outcomes observed in *T. brucei* MSH2 mutants generated in PCFs may be explained by the greater burden of oxidative stress that the parasites are submitted during their insect life cycle stages (Grazielle-Silva et al., 2015).

Here we asked whether the role played by *T. brucei* and *T. cruzi* MSH2 in the oxidative stress response is MMR-independent or if MSH2 acts together with other MMR proteins in a non-canonical pathway. Toward that, we generated *T. brucei* BSF mutants with deleted *msh3* or *msh6*, and *T. cruzi* epimastigotes *msh6* null mutants. As indicated above, studies with *T. brucei mlh1* knockout cell lines have already indicated that this MMR protein is not involved in the oxidative stress response (Grazielle-Silva et al., 2015). Initially, co-immunoprecipitation assays followed by mass spectrometry showed that *T. brucei* MSH2 physically interacts with MSH3 and MSH6 in both BSF and PCF forms, though no other proteins were identified in the co-immunoprecipitation complex that contains MSH2. We also showed that, MSH2 interacts with MSH6 in *T. cruzi* epimastigotes. We have repeated the co-immunoprecipitation assay after treatment with MNNG or H_2_*O*_2_, but no novel interactions with MSH2 could be identified (data not shown).

In contrast to *msh3* knockout parasites, *T. brucei msh6* null mutants, but not the heterozygous mutants, showed impaired MMR, since tolerance to MNNG was increased in *msh6*-/- BSFs compared to WT BSF, similar to previous observation in BSF *msh2*-/- mutants (Bell and McCulloch, 2003). O^6^meG lesions can be formed after DNA alkylation by MNNG and attempts to repair this damage leads to futile repair cycles that ends up signaling or causing cell death (Gupta et al., 2018). A similar phenotype was observed with *T. cruzi msh6* null mutants after MNNG treatment. These results thus indicate that the absence of MSH6 results in non-functional MMR in both parasites. Although we showed that MSH3 is a binding partner to MSH2 in both *T. brucei* BSFs and PCFs, MSH3 does not seem to be involved in the response to this alkylating damage. What substrates the *T. brucei* MSH2-MSH3 heterodimer acts on remain to be determined.

After showing evidence that the lack of MSH6 affects the MMR pathway in both parasites, we showed that in *T. brucei* BSFs, MSH3, and MSH6 seems not to be involved in the oxidative stress response, using the same experimental conditions applied previously with *msh2* null mutants. The effect of disrupting *msh3* and *msh6* genes in PCFs still needs to be verified. Also, to test the hypothesis that *T. brucei* MSH2 is involved in the response to oxidative DNA damage in a way that is independent of other MMR components, a double mutant *Tbmsh6*-/-*msh3*-/- needs to be generated to compare the differences in the response to H_2_*O*_2_ treatment with WT parasites. With a double *msh3/msh6* mutant, we would be able to exclude the possibility that, in the absence of MSH6, MSH2 may still form a complex with MSH3 and provide a role in tackling oxidative stress.

Nevertheless, the lack of sensitivity of *msh6* null mutants to oxidative damage adds detail to our hypothesis that MSH2 may provide a unique function in this role, as initially shown by our previous studies showing that *T. brucei* BSF *msh2* null mutants complemented with *T. cruzi msh2* have restored their capacity to respond to oxidative stress response, but have not restored MMR function (Machado-Silva et al., 2008).

The results obtained with the analyses of *T. brucei* mutants are in sharp contrast with the effect of disrupting the *msh6* gene in *T. cruzi* epimastigotes, which showed a clear effect on the susceptibility to H_2_*O*_2_ treatment. Besides increased susceptibility to oxidative stress, *T. cruzi msh6*-/- mutants display increased resistance to MNNG, suggesting impaired MMR. Although we were able to demonstrate that re-expressing MSH6 causes the sensitivity to H_2_*O*_2_ to be reverted, we do not see a significant reversion of the tolerance to MNNG, which may due to an imbalanced expression of MSH6 and altered dynamics of MSH2-MSH6 function during MMR. The effect observed with *T. cruzi msh6* mutants is in line with the expected role of MSH6 as a MSH2 partner during the initial steps of the MMR. However, because in *T. cruzi* epimastigotes *msh2* knockouts presented an increased tolerance to H_2_*O*_2_ treatment, whereas MSH6 deficient cells were more sensitive to the same treatment, the role of MSH6 as a MSH2 partner in the response to oxidative stress is unclear. The fact that re-expression of MSH6 in the *msh6* knockout mutants reverts the H_2_*O*_2_ susceptibility further corroborates its role as a component of the DNA repair machinery involved with the oxidative stress response. It is possible that the generation of *T. cruzi msh6* deletions with the highly efficient CRISPR/Cas9 protocol makes it impossible for an adaptation process that we speculated that may have resulted from the *msh2* disruption obtained with the classical HR-based protocol, which has a slower selection of drug resistant parasites. Thus, in contrast to the *msh2* mutants, the rapid generation of *msh6*-/- may not have allowed the mutated parasites to adapt their metabolism to cope with the oxidative stress response in the absence of a MMR protein (Grazielle-Silva et al., 2015). Recent evidence suggests that an oxidative environment is favorable for intracellular parasite survival and infection (Paiva et al., 2018). The parasite has an arsenal of enzymes to assure infection and keeps homeostasis while going into different redox environments inside the hosts (Mesías et al., 2019). How this plasticity is achieved is still unclear. Long-term cultivation of *T. cruzi* induces mutational events. This frequency is increased when parasites are cultivated in sub-lethal doses of H_2_*O*_2_ (Torres-Silva et al., 2018). In trypanosomatids, MMR proteins might have an additional role in recognizing DNA oxidative damage and act as signaling molecules. It is possible that after long-term cultivation of *T. cruzi msh6*-/- in sub-lethal doses of H_2_*O*_2_ parasites adapted to the oxidative stress, such as *T. cruzi msh2*-/- could be obtained. It should be also considered that, since MSH2 forms pairs with MSH3 or MSH6, the formation of the heterodimer may be required for the stability of MSH2 and loss of MSH6 could lead to a destabilization and a direct impact on the levels of MSH2 protein. Consequently, in a model where the oxidative damage response mechanism requires MSH2 independently of other MMR proteins, the lack of MSH6 may have an indirect effect by reducing MSH2 half-life. Taken together, our data suggest that MSH2, but not MSH6, has a predominant role in the oxidative stress response in *T. cruzi*. The observation showing no differences in the survival rates after infection of two different cell types with *T. cruzi msh6*-/-, whereas a clear difference was observed when macrophages were infected with *msh2* mutants compared to WT parasites (Grazielle-Silva et al., 2015), also points toward a distinct role between these two MMR components in the oxidative stress response of *T. cruzi*.

With the current data, one cannot conclude whether *T. cruzi* MSH6 acts together with MSH2 to recognize oxidative damage, or if MSH6 acts by maintaining the stability of the MutSα heterodimer, which may be also important for *T. brucei* MSH2. In a scenario of a non-canonical MMR function in the oxidative stress response it is also possible that, even though MSH6 in *T. cruzi* is also involved in the oxidative stress response, by recognizing DNA damage through MutSα heterodimer, MSH2 may have a more predominant role with the recruitment of proteins related to damage signaling, such as ATR (Wang and Qin, 2003; Stojic et al., 2004; Yan et al., 2014). In *Leishmania major* has been demonstrated that ATR is involved in the oxidative stress response (da Silva et al., 2018). Clearly, the involvement of other signaling molecules in the oxidative stress response in *T. cruzi* and *T. brucei* needs to be further investigated. Although it is not surprising that the oxidative stress response works differently in *T. cruzi* and *T. brucei*, since these parasites have diverged around 100 million years ago (Stevens et al., 1999), the additional studies needed to clarify this response in these two important human pathogens may still bring new interesting surprises.

## Data Availability Statement

The raw data supporting the conclusions of this article will be made available by the authors, without undue reservation, to any qualified researcher.

## Ethics Statement

The animal study was reviewed and approved by Comissão de Ética no USO de Animais - CEUA - UFMG.

## Author Contributions

VG-S, TZ, CM, RM, and ST conceived and designed the experiments. VG-S, TZ performed the experiments. VG-S, TZ, RB, CM, RM, and ST analyzed the data. RB, CM, RM, and ST contributed reagents, materials and analysis tools. VG-S, RM and ST wrote the paper.

### Conflict of Interest

The authors declare that the research was conducted in the absence of any commercial or financial relationships that could be construed as a potential conflict of interest.
