# Supplementary material for: Trypanosoma brucei and Trypanosoma cruzi DNA Mismatch Repair Proteins Act Differently in the Response to DNA Damage Caused by Oxidative Stress
Source: Front Cell Infect Microbiol. 2020 Apr 16;10:154. doi: 10.3389/fcimb.2020.00154 (PMC7176904; doi:10.3389/fcimb.2020.00154)
Supplement: Supplementary file 1 [file Data_Sheet_1.zip › Figure S1.pdf]

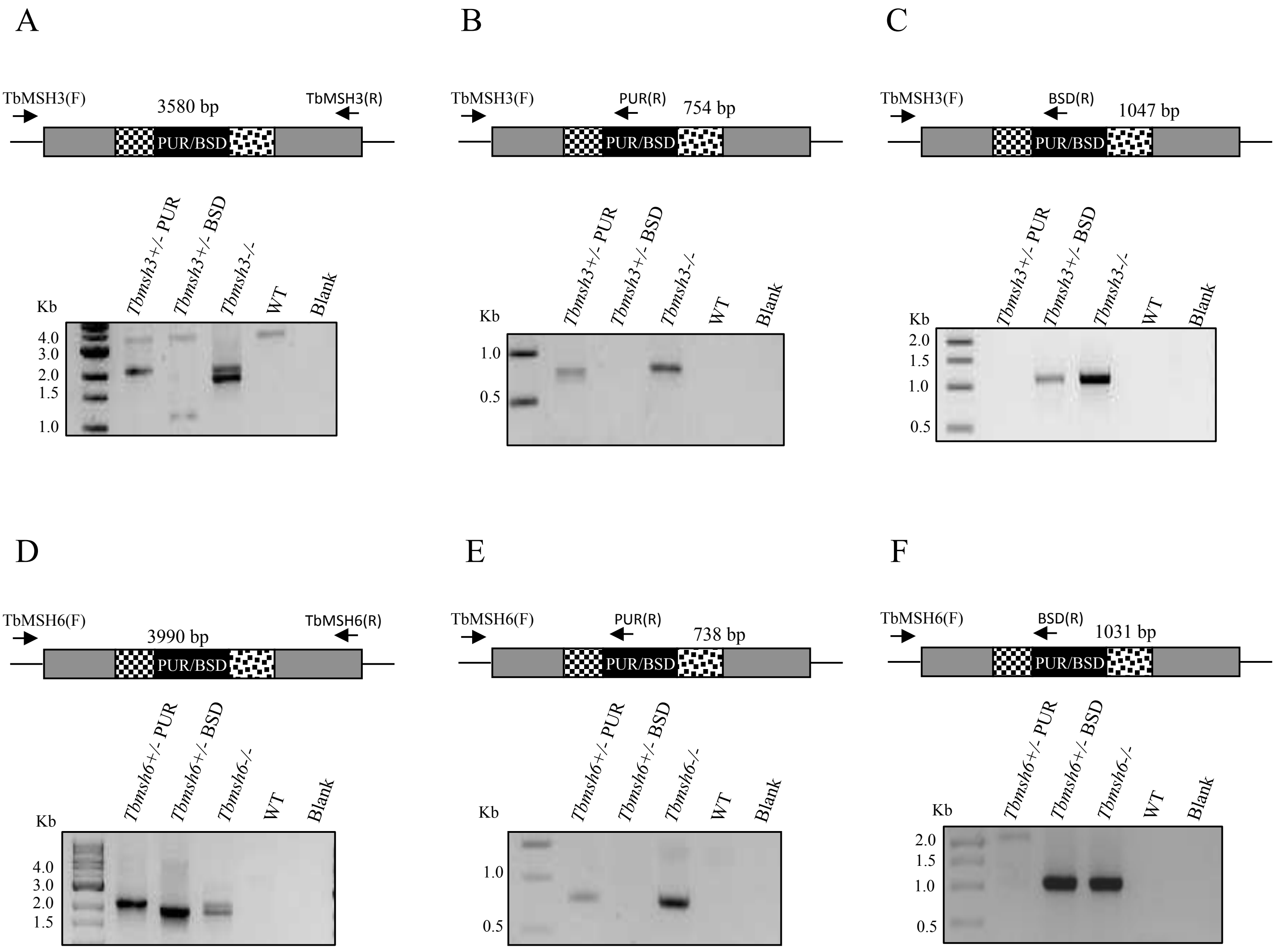

Supplementary Figure 1: Confirmatory PCR for *T. brucei* MSH3 and MSH6 knockouts. Different sets of primers (denoted by arrows and labelled) were designed to test the organisation of the *msh3* (A, B and C) and *msh6* (D, E and F) locus in the WT, *Tbmsh3*+/-BSD, *Tbmsh3*+/-PUR and *Tbmsh3*-/- cells: gels are shown of PCR with these primers on genomic DNA from the cells; blank denotes a reaction with no substrate DNA. (**A**) Primers TbMSH3(F) and TbMSH3(R) were used to PCR-amplify the ORF of *Tbmsh3*. (**B**) In an attempt to confirm the presence of the puromycin (PUR) resistance cassette, PCR was performed using TbMSH3(F) as forward primer and a reverse primer complementary to PUR (PUR (R)). (**C**) To test for the Blasticidin resistance cassette (BSD) PCR was performed was using TbMSH3(F) as the forward primer and a reverse primer complementary to BSD (BSD (R)). (**D**) Primers TbMSH6(F) and TbMSH6(R) were used to PCR-amplify the ORF of *Tbmsh6*. (**E**) In an attempt to confirm the presence of the puromycin (PUR) resistance cassette, PCR was performed using TbMSH6(F) as forward primer and a reverse primer complementary to PUR (PUR (R)). (**F**) To test for the Blasticidin resistance cassette (BSD) PCR was performed was using TbMSH6(F) as the forward primer and a reverse primer complementary to BSD (BSD (R)).
