# Supplementary material for: Trypanosoma brucei and Trypanosoma cruzi DNA Mismatch Repair Proteins Act Differently in the Response to DNA Damage Caused by Oxidative Stress
Source: Front Cell Infect Microbiol. 2020 Apr 16;10:154. doi: 10.3389/fcimb.2020.00154 (PMC7176904; doi:10.3389/fcimb.2020.00154)
Supplement: Supplementary file 1 [file Data_Sheet_1.zip › Figure S2.PDF]

A

```
                                   20                    40                    60                    80
     TritrypDB ATGGATGACG CTCTAGTTGA CTGTGCGGAC GTTTACCGCA TGGAATACAC CTATTCTTTC TTGGCGGGAA TAGATCCTAT 80
  Non-Esmeraldo ATGGATGACG CTCTAGTTGA CTGTGCGGAC GTTTACCGCA TGGAATACAC CTATTCTTTC TTGGCGGGAA TAGATCCTAT 80
     Esmeraldo ATGGATGACG CCCTAGTTGA CTGTGCGGAC GTTTACCGCA TGGAATACAC CTATTCTTTC TTGGCGGGAA TAGATCCTAT 80
                                  100                   120                   140                   160
     TritrypDB GCGGCCGCCC TCGTCGATTA CGATCCCCGC CAAGGAACTG GACGCCATGG CGGGCATGGA GCGGCAGTAC TGGGAGATCA 160
  Non-Esmeraldo GCGGCCGCCC TCGTCGATTA CGATCCCCGC CAAGGAACTG GACGCCATGG CGGGCATGGA GCGGCAGTAC TGGGAGATCA 160
     Esmeraldo GCGGCCGCCC TCGTCGATTA CGATCCCCGC CAAGGAACTG GACGCCATGG CGTGCATGGA GCGGCAGTAC TGGGAGATCA 160
                                  180                   200                   220                   240
     TritrypDB AGGCAAAGTA CTTTGATGTG TTAATTCTCT TTAAAAAGGG AAAGTTTTAT GAGCTGTACG ATCAAGACGC CGCCATCGCA 240
  Non-Esmeraldo AGGCAAAGTA CTTTGATGTG TTAATTCTCT TTAAAAAGGG AAAGTTTTAT GAGCTGTACG ATCAAGACGC CGCCATCGCA 240
     Esmeraldo AGGCAAAGTA CTTTGATGTG TTAATTCTCT TTAAAAAGGG AAAGTTTTAT GAGCTGTACG ATCAAGACGC TGCCATCGCA 240
                                  260                   280                   300                   320
     TritrypDB CACCGCGAAT TTGGCCTCAA GCTTGTTTTT AGCGCCACCA ACCGTGGCAA GATGCGTCTT GCGGGGGTTC CAGAGCAAAG 320
  Non-Esmeraldo CACCGAGAAT TTGGCCTCAA GCTTGTTTTC AGCGCCACCA ACCGTGGCAA GATGCGTCTT GCGGGGGTTC CAGAGCAAAG 320
     Esmeraldo CACCGCGAAT TTGGCCTCAA GCTTGTTTTT AGCGCCACCA ACCGTGGCAA GATGCGTCTT GCGGGGGTTC CAGAGCAAAG 320
                                  340                   360                   380                   400
     TritrypDB CTTCAGCGAG TGGGCACGCC TTTTTGTCTT CCGCGGCTAC AAGGTGGGCA GAGTAGAACA AATGAAGGAG GAAACGGATA 400
  Non-Esmeraldo CTTCAGCGAA TGGGCACGCC TTTTTGTCTT CCGCGGCTAC AAGGTGGGCA GAGTAGAACA AATGAAGGAG GAAACGGATA 400
     Esmeraldo CTTCAGCGAG TGGGCACGCC TTTTTGTCTT CCGCGGCTAC AAGGTGGGCA GAGTAGAACA AATGAAGGAG GAAACGGATA 400
                                  420                   440                   460                   480
     TritrypDB CCTCCCCTAT CAAGACTGCA CGCACGAAGG TGCTACAACG TGAGTTGGTT GAAGTGCTCA CACCAGGCAC ATTGACAGAT 480
  Non-Esmeraldo ACTCCCCTAT CAAGACTGCA CGCACGAAGG TGCTACAACG TGAGTTGGTT GAAGTGCTCA CACCAGGCAC ATTGACAGAT 480
     Esmeraldo CCTCCCCTAT CAAGACTGCA CGCACGAAGG TGCTACAACG TGAGTTGGTT GAAGTGCTCA CACCAGGCAC ATTGACAGAT 480
                                  500                   520                   540                   560
     TritrypDB CCTGCAATGC TTAGCGGTTC CGGTCCTGTC TTTATTCTGG CCCTCTGTCC CTTGCAGGAA AATGTTGTGG ACGGTTTGGC 560
  Non-Esmeraldo CCTGCAATGC TTAGCGGTTC CGGTCCTGTC TTTATTCTGG CCCTCTGTCC CTTGCAGGAA AATGTTGTGG ACGGTTTGGC 560
     Esmeraldo CCTGCAATGC TTAGCGGTTC CGGTCCTGTC TTTATTCTGG CCCTCTGTCC CTTGCAGGAA AATGTTGTGG ACGGTTTGGC 560
                                  580                   600                   620                   640
     TritrypDB GGTGGATCTC TCACGTCATG TGGTTTATCA CTGTCCATGT GGCGTCGGGA GAAATGACGT GTTGCGTGAG GAGGAGACGC 640
  Non-Esmeraldo GGTGGATCTC TCACGTCATG TGGTTTATCA CTGTCCATGT GGCGTCGGGA GAAATGACGT GTTGCGTAAG GAGGAAACGC 640
     Esmeraldo GGTGGATCTC TCACGTCATG TGGTTTATCA CTGTCCATGT GGCGTCGGGA GAAATGACGT GTTGCGTGAG GAGGAGACGC 640
                                  660                   680                   700                   720
     TritrypDB TGCTCATGGT GTGTGCCCTC TTGCAGCAGC TCCGCCCGCG TGAAATCATA TTTCCACTTT GTTTTATTCC TAATGCTGCG 720
  Non-Esmeraldo TGCTCATGGT GTGTGCCCTC TTGCAGCAGC TCCGCCCGCG TGAAATCATA TTTCCACTTT GTTTTGTTCC TAATGCTGCG 720
     Esmeraldo TGCTCATGGT GTGTGCCCCC TTGCAGCAGC TCCGCCCGCG TGAAATCATA TTTCCACTTT GTTTTATTCC TAATGCTGCG 720
                                  740                   760                   780                   800
     TritrypDB CAGGCCGATA GGAAGGCATC ATTTGCCAAG CGTTTGGTGG ATTGGATAGA GGGCGAGGGG TTTCGGGTGG AGTTGGTGGA 800
  Non-Esmeraldo CAGGCCGATA GGAAGGCATC ATTTGCCCAG CGTTTGTTGG ATTGGATAGA GGGCGAGGGG TTTCGGGTGG AGTTGGTGGA 800
     Esmeraldo CAGGCCGATA GGAAGAAATC ATTTGCCAAG CGTTTGGTGG ATTGGATAGA GGGCGAGGGG TTTCGGGTGG AGTTGGTGGA 800
                                  820                   840                   860                   880
     TritrypDB TGTGTCTTCT CTCTCGTTTT CAAAAGGCAA CTCCGATGGG GTGGACAAAA TTTTGGACGC GCACAAGTTC TTGGCACACT 880
  Non-Esmeraldo TGTGTCTTCT CTCTCGTTTT CGAAAGGCAA CTCCGATGGG GTGGACAAAA TTTTGGACGC GCACAAGTTC TTGGCACACT 880
     Esmeraldo TGTGTCTTCT CTCTCGGTTT CAAAAGGCAA CTCCGATGGG GTGGACAAAA TTTTAGACGC GCACAAGTTC TTGGCACACT 880
                                  900                   920                   940                   960
     TritrypDB ACTTTCAGAC GTTGAAACTT TACCATGCCG TGCCCATACT CTCAGAGGCA GAACCCTACA CCTTCCATCT TCCTTACGCA 960
  Non-Esmeraldo ACTTTCAGAC GTTGAAACTT TACCATGCCG TGCCCATACT CTCAGAGGCA GAACCCTACA CCTTCCATCT TCCTTACGCA 960
     Esmeraldo ACTTTCAGAC GTTGAAACTT TACCATGCCG TGCCCATACT CTCAGATGCA GAACCCTACA CCTTCCATCT TCCTTACGCA 960
                                  980                 1.000                 1.020                 1.040
     TritrypDB TCCTCCAATA CCACCATTGC TCGCAAGCAT GAGGGTGAAG TTTCAAATTC TTCATCGATA TTATGGCATG AACGAAGAGT 1040
  Non-Esmeraldo TCCTCCAATA CCACCATTGC TCGCAAGCAT GAGGGTGAAG TTTCAAATTC TTCATCGATA TTATGGCATG AACGAAGAGT 1040
     Esmeraldo TCCTCCAATA CCGCCATTGC TCGCAAGCAT GAGGGTGGGG TTTTAAATTC TTCATCGATA TTATGGCATG AACGAAGAGT 1040
                                1.060                 1.080                 1.100                 1.120
     TritrypDB GGACCGTGGA CTCGTGCTGG ATGCCACAAC CGTGAGCAAC TTGGAGCTAG TCAGCAACTT GCGGGATGGC GGGGAACGGG 1120
  Non-Esmeraldo GGACCGTGGA CTCGTGCTGG ATGCCACAAC CGTGAGCAAC TTGGAGCTAG TCAGCAACTT GCGGGATGGC GGGGAACGGG 1120
     Esmeraldo GGACCGTGGA CTCGTGCTGG ATGCCACAAC CGTGAGCAAC TTGGAGCTGG TCAGCAACTT GCGGGATGGC GGGGAACGGG 1120
                                1.140                 1.160                 1.180                 1.200
     TritrypDB GCTCACTGAA TCAACTTTTC AATCGCTGCT GTACAAATGG AGGAAAGCGT CTCATGCGAT CATGGATTTT ACGGCCTTCT 1200
  Non-Esmeraldo GCTCACTGAA TCAACTTTTC AATCGCTGCT GTACAAATGG AGGAAAGCGT CTCATGCGAT CATGGATTTT ACGGCCTTCT 1200
     Esmeraldo GCTCACTGAA TCAACTGTTC AATCGCTGCT GTACAAATGG AGGAAAGCGT CTCATGCGAT CATGGATTTT ACGGCCTTCT 1200
                                1.220                 1.240                 1.260                 1.280
     TritrypDB GCTTCCTCCC GTGTCATTCT GGCTCGACAG GAGGCGATTC GCTTCATTAT TGAGCACAAG CTTGACGAGC TTTGGGGCGA 1280
  Non-Esmeraldo GCTTCCTCCC GTGTCATTCT GGCTCGACAG GAGGCGATTC GCTTCATTAT TGAGCACAAG CTTGACGAGC TTTGGGGCGA 1280
     Esmeraldo GCTTCCTCCC GTGTCATTCT GGCTCGACAG GAGGCGATTC GCTTCATTAT TGAGCACAAG CTTGACGAGC TTTGGGGCGA 1280
                                1.300                 1.320                 1.340                 1.360
     TritrypDB GGGTGGAGAG CCGGAGACGA CGTTGGGGAT TGCAGCGGCG TCCCCCACAC TGAGAGCAGA AACCCCTCGT GAGCATGAAC 1360
  Non-Esmeraldo GGGTGGAGAG CCGGAGACGA CGTTGGGGAT TGCAGCGGCG TCCCCCACAC TGAGAGCAGA AACCCCTCGT GAGCATGAAC 1360
     Esmeraldo GGGTGGAGAG CCGGAGACGA CGTTGGGGAT TGCAGCGGCG CCCCCCACAG TGAGAGCAGA AACCCCTCGT GAGGATGAAC 1360
                                1.380                 1.400                 1.420                 1.440
     TritrypDB CAATTTGTGT TGTGAAACGA GAGCGGCCGG TGGGTAGTAA ATTTGAAACA CGTTTCTCCA GTTTGGTGGA TGTGGATTTT 1440
  Non-Esmeraldo CAATTTGTGT TGTGAAACGA GAGCGGCCGG TGGGTAGTAA ATTTGAAACA CGTTTCTCCA GTTTGGTGGA TGTGGATTTT 1440
     Esmeraldo CAAGTTGTGT TGTGAAACGA GAGCGGCCGG TGGGTAGTAA ATTTGAAACA CGTTTCTCCA GTTTGGTGGA TGTGGATTTT 1440
                                1.460                 1.480                 1.500                 1.520
     TritrypDB GAGCGTAATC TTTCTCGCTT GACGGACTTG AAGAACAACA GTGACGCACA AGTGGCGTTT GTTGATCCCC TTGTTCAGTA 1520
  Non-Esmeraldo GAGCGTAATC TTTCTCGCTT GACGGACTTG AAGAACAACA GTGACGCACA AGTGGCGTTT GTTGATCCCC TTGTTCAGTA 1520
     Esmeraldo GAGCGTAATC TTTCTCGCTT GACGGACTTG AAGAGCAACA GTGACGCACA AGTGGCGTTT GTTGATCCCC TTGTTCAGTA 1520
                                1.540                 1.560                 1.580                 1.600
     TritrypDB CAAGAAGCAA CTTCAAATCA TTGTTACAAC GGTCCAGGCT CTTGAGGACA TGGTGGCATG GTCACACGAC ATACAAAAGG 1600
  Non-Esmeraldo CAAGAAGCAA CTTCAAATCA TTGTTACAAC GGTCCAGGCT CTTGAGGACA TGGTGGCATG GTCACACGAC ATACAAAAGG 1600
     Esmeraldo CAAGAAGCAA CTTCAAATCA TTATTACAAC GGTCCAGGCT CTTGAGGACA TGGTGGCATG GTCACACGAC ATACAAAAGG 1600
```

```
                                    1.620                                   1.640                                   1.660                                   1.680
TritrypDB     GGATAACCTC GTCACCGCCT CTTTTGAAGG AGCTCTGGGC GCAAATTGAT GCTGCCGCTC CCGCGGTAAC CTCCATTGGA 1680
Non-Esmeraldo GGATAACCTC GTCACCGCCT CTTTTGAAGG AGCTCTGGGC GCAAATTGAT GCTGCCGCTC CCGCGGTAAC CTCCATTGGA 1680
Esmeraldo     GGATAACTTC GTCACCACCT CTTTTGAAGG AGCTCTGGGC GCAAATTGAT GCTGCCGCTC CCGCGGTAAC CTCCATTGGA 1680
                                    1.700                                   1.720                                   1.740                                   1.760
TritrypDB     AACTGTTTTG ACCGCCATGC TGCCCTTGCG TCGGGCGTGA TTGTTCCCTC TCAGGGAGCA TCCTCCGTGT ATGATGAGGC 1760
Non-Esmeraldo AACTGTTTTG ACCGCCATGC TGCCCTTGCG TCGGGCGTGA TTGTTCCCTC TCAGGGAGCA TCCTCCGTGT ATGATGAGGC 1760
Esmeraldo     AACTGTTTTG ACCGCCATGC TGCCCTTGCG TCGGGCGTGA TTGTTCCCTC TCAGGGAACA TCCTCCGTGT ATGATGAGGC 1760
                                    1.780                                   1.800                                   1.820                                   1.840
TritrypDB     ATCCGGTACT CTCGACGCCA TTGAGGGAAA ATTACAGGGG GAACTACGCC GATTACAAGA GGAGGTTTTT GATGGTACAG 1840
Non-Esmeraldo ATCCGGTACT CTCGACGCCA TTGAGGGAAA ATTACAGGGG GAACTACGCC GATTACAAGA GGAGGTTTTT GATGGTACAG 1840
Esmeraldo     ATCCGGTACT CTCGACGCCA TTGAGGGAAA ATTACAGGGG GAATTACGCC GACTACAAGA GGAGGTTTTT GATGGTGCAG 1840
                                    1.860                                   1.880                                   1.900                                   1.920
TritrypDB     CGATCAACTA CAGCGTCGTT GGTCACGAGC AATTTCTTGT AGAAGTGCCA ATTTCTGCTG TACCGAAAAC GCCTTTGCGT 1920
Non-Esmeraldo CGATCAACTA CAGCGTCGTT GGTCACGAGC AATTTCTTGT AGAAGTGCCA ATTTCTGCTG TACCGAAAAC GCCTTTGCGT 1920
Esmeraldo     CGATCAACTA CAGCGTCGTT GGTCACGAGC AATTTCTTGT AGAGGTGCCA ATTTGTGCTG TACCGAAAAC GCCTTTGCGT 1920
                                    1.940                                   1.960                                   1.980                                   2.000
TritrypDB     GGATTTGTGG AAAGATCCCG CAGTGGCAAG AGTGTGAAGT ATGTTGTGGC CTCCCTGGAG CCACTGGTGG AGTCTCATAA 2000
Non-Esmeraldo GGATTTGTGG AAAGATCCCG CAGTGGCAAG AGTGTGAAGT ATGTTGTGGC CTCCCTGGAG CCACTGGTGG AGTCTCATAA 2000
Esmeraldo     GGATTTGTGG AACGATCCCG CAGTGGCAAG AGTGTGAAGT ATGTTGTGGC CGCCCTGGAG CCACTGGTGG AGGCTCATAA 2000
                                    2.020                                   2.040                                   2.060                                   2.080
TritrypDB     GAAAGCAAAG AAGGAAAAAT CAAACGCTCT TCTTTTGGTG CTGCAAAATG TTGCATCGCA CATGTGTCAC TACTTCCCTC 2080
Non-Esmeraldo GAAAGCAAAG AAGGAAAAAT CAAACGCTCT TCTTTTGGTG CTGCAAAATG TTGCATCGCA CATGTGTCAC TACTTCCCTC 2080
Esmeraldo     GAAAGCAAAG AAGGAAAAGT CAAACGCTCT TCTTTTGGTG CTGCAAAATG TTGCATCGCA CATGTGTCAC TACTTCCCTC 2080
                                    2.100                                   2.120                                   2.140                                   2.160
TritrypDB     TCCTGTATGG TGCCACGGAG GCACTCTCAT ACTTTGATTG CCTCTTGAGT CTTGCGTCAC TGCGAAAATG TGGGGTCACA 2160
Non-Esmeraldo TCCTGTATGG TGCCACGGAG GCACTCTCAT ACTTTGATTG CCTCTTGAGT CTTGCGTCAC TGCGAAAATG TGGGGTCACA 2160
Esmeraldo     TCCTGTACGG TGCCACGGAG GCACTCTCAT ACTTTGATTG CCTCTTGAGT CTTGCGTCAC TGCGAAAATG TGGGGTCACA 2160
                                    2.180                                   2.200                                   2.220                                   2.240
TritrypDB     ACCGCTTGGC CTATTGTGAA GGATGACGCT GTGGGGGCGA GTCTGGAGGC CGAGCAACTA ACTCATCCTT TTCTAAATGG 2240
Non-Esmeraldo ACCGCTTGGC CTATTGTGAA GGATGACGCT GTGGGGGCGA GTCTGGAGGC CCGGGAACTA ACTCATCCTT TTCTAAATAG 2240
Esmeraldo     ACCGCTTGGC CTATTGTGAA GGATGACGCT GTGGGGGCGA GTCTGGAGGC CGAGCAACTA ACTCATCCTT TTCTAAATGG 2240
                                    2.260                                   2.280                                   2.300                                   2.320
TritrypDB     AGACTCTGTG CCGAACGATA TTTCCTTGGA CGCGGCGCAC GGGCGTATTT TGCTCTTGAC GGGGCCGAAC ATGGCGGGGA 2320
Non-Esmeraldo AGACTCTGTG CCGAACGATA TTTCCTTGGA CGCGGCGCAC GGGCGTATTT TGCTCTTGAC GGGGCCGAAC ATGGCGGGGA 2320
Esmeraldo     AGACTCTGTG CCGAACGATA TTTCCTTGGA CGCGGCGCAC GGGCGTATTT TGCTCTTGAC GGGGCCGAAC ATGGCGGGGA 2320
                                    2.340                                   2.360                                   2.380                                   2.400
TritrypDB     AAAGTACACT TATGCGTACG GTTGCTGTGA ACGTCATCAT TGCGCAAATG GGAGGACCAA TTTTTGGTGC ATTTATGCGT 2400
Non-Esmeraldo AAAGTACACT TATGCGTACG GTTGCTGTGA ACGTCATCAT TGCGCAAATG GGAGGACCAA TTTTTGGTGC ATTTATGCGT 2400
Esmeraldo     AAAGTACACT TATGCGTACG GTTGCTGTGA ACGTCATCAT TGCGCAAATG GGGGGACCAA TTTTTGGTGC ATTTATGCGT 2400
                                    2.420                                   2.440                                   2.460                                   2.480
TritrypDB     CTTGCTACGG TGTCCCGCAT CTTCACACGT ATCGGAGCCC GCGATGCTTC TCACAAGGGA CAGAGTACAC TGTATGTTGA 2480
Non-Esmeraldo CTTGCTACGG TGTCCCGCAT CTTCACACGT ATCGGAGCCC GCGATGCTTC TCACAAGGGA CAGAGTACAC TGTATGTTGA 2480
Esmeraldo     CTTGCTACGG TGTCCCGCAT CTTCACACGT ATCGGAGCCC GCGATGCTTC TCACAAGGGA CAGAGTACAC TGTATGTTGA 2480
                                    2.500                                   2.520                                   2.540                                   2.560
TritrypDB     GCTGAGCGAA ACAGCTGACA TCCTTCGTCA TGCAGACCCA TGGAGTTTAT GTCTGGTAGA TGAATTGGGG CGTGGTACCT 2560
Non-Esmeraldo GCTGAGCGAA ACAGCTGACA TCCTTCGTCA TGCAGACCCG TGGAGTTTAT GTCTGGTAGA TGAATTGGGA CGTGGTACCT 2560
Esmeraldo     GCTGAGCGAA ACAGCTGACA TCCTTCGTCA TGCAGACCCA TGGAGTTTAT GTCTGGTAGA TGAATTGGGG CGTGGTACCT 2560
                                    2.580                                   2.600                                   2.620                                   2.640
TritrypDB     CTACACATGA CGGTTACGCC ATTGCACATG CCACATTACA CTCGTTGAAG GAACGCCTTC CTGTTTCCCC GCTTCTCCTG 2640
Non-Esmeraldo CTACACATGA CGGTTACGCC ATTGCACATG CCACATTACA CTCGTTGAAG GAACGCCTTC CTGTTTCCCC GCTTCTCCTG 2640
Esmeraldo     CTACACATGA CGGTTACGCC ATTGCACATG CCACATTACA CTCGTTGAAG GAACGCCTTC CTGTTTCCCC GCTTCTCCTG 2640
                                    2.660                                   2.680                                   2.700                                   2.720
TritrypDB     TTTTCCACCC ACTATCACGC TCTTGCACAG GAACAGCTTG GTGGGGATGC CGTGAACGTA TCGTCTTTGG CGCGAGATCA 2720
Non-Esmeraldo TTTTCTACCC ACTATCACGC TCTTGCACAG GAACAGCTTG GCGGGGATGC CGTGAACGTA TCGTCTTTGG CCCCAGATCA 2720
Esmeraldo     TTTTCCACCC ACTATCACGC TCTTGCACAG GAACAGCTTG GTGGGGATGC CGTGAACGTA TCGTCTTTGG CGCGAGATCA 2720
                                    2.740                                   2.760                                   2.780                                   2.800
TritrypDB     TGGCTCGGTG GTGCAGCTGG GATACATGGA CTTTGCTATA TCGGATACAA AAAAAGATGG TGTTTCGGCA ATTACATTTC 2800
Non-Esmeraldo TGGCTCGGTG GTGCAGTTGG GATACATGGA CTTTGCTATA TCGGATACAA AAAAAGATGG TGTTTCGGCA ATTACATTTC 2800
Esmeraldo     TGGCTCGGTG GTGCAGCTGG GATACATGGA CTTTGCTATA TCGGATACAA AAAAAGATGG TGTTTCGGCA ATTACATTTC 2800
                                    2.820                                   2.840                                   2.860                                   2.880
TritrypDB     TTTACCGTCT TGTCTCTGGC GTATGCACAC GTAGCTATGG CGTTGAGGTG GCTTTACTGG CTGGCATTCC TTCTTCACTC 2880
Non-Esmeraldo TTTACTGTCT CGTCTCTGGC ATATGCACAC GTAGCTATGG CGTTGAGGTG GCTTTACTGG CTGGCATTCC CTCTTCACTC 2880
Esmeraldo     TTTACCGTCT TGTCTCTGGC GTATGCACAC GTAGCTATGG CGTTGAGGTG GCTTTACTGG CTGGCATTCC TTCTTCACTC 2880
                                    2.900                                   2.920                                   2.940                                   2.960
TritrypDB     GTGCACATGG CAGCCATCAA GTCACATGAG CTTGCCTCAT GGAATGACCG GCAAAAGGAC ATTCATACCA TACGACAGTT 2960
Non-Esmeraldo GTGCACATGG CAGCCATCAA GTCACATGAG CTTGCCTCAT GGAATGACCG GCAAAAGGAC ATTCATACCA TACGACAGTT 2960
Esmeraldo     GTGCACATGG CAGCCATCAA GTCACATGAG CTTGCCTCAT GGAATGACCG GCAAAAGGAC ATTCATACCA TACGACAGTT 2960
                                    2.980                                   3.000
TritrypDB     CCTTCATGAG CCTAACGCTG CCACACTTTT CAAGAAAAAG AAGTCGTAG 3009
Non-Esmeraldo CCTTCATGGG CCTAACGCTG CCACACTTTT CAAGAAAAAG AAGTCGTAG 3009
Esmeraldo     CCTTCATGAG CCTAACGCTG CCACACTTTT CAAGAAAAAG AAGTCGTAG 3009
```

B

```
                                      20                                       40                                       60                                       80
TritrypDB  MDDALVDCAD VYRMEYTYSF LAGIDPMRPP SSITIPAKEL DAMAGMERQY WEIKAKYFDV LILFKKGKFY ELYDQDAAIA HREFG 85
  Non-Esm  MDDALVDCAD VYRMEYTYSF LAGIDPMRPP SSITIPAKEL DAMAGMERQY WEIKAKYFDV LILFKKGKFY ELYDQDAAIA HREFG 85
      Esm  MDDALVDCAD VYRMEYTYSF LAGIDPMRPP SSITIPAKEL DAMACMERQY WEIKAKYFDV LILFKKGKFY ELYDQDAAIA HREFG 85
                                 100                                      120                                      140                                      160
TritrypDB  LKLVFSATNR GKMRLAGVPE QSFSEWARLF VFRGYKVGRV EQMKEETDTS PIKTARTKVL QRELVEVLTP GTLTDPAMLS GSGPV 170
  Non-Esm  LKLVFSATNR GKMRLAGVPE QSFSEWARLF VFRGYKVGRV EQMKEETDNS PIKTARTKVL QRELVEVLTP GTLTDPAMLS GSGPV 170
      Esm  LKLVFSATNR GKMRLAGVPE QSFSEWARLF VFRGYKVGRV EQMKEETDTS PIKTARTKVL QRELVEVLTP GTLTDPAMLS GSGPV 170
                      180                                      200                                      220                                      240
TritrypDB  FILALCPLQE NVVDGLAVDL SRHVVYHCPC GVGRNDVLRE EETLLMVCAL LQQLRPREII FPLCFIPNAA QADRKASFAK RLVDW 255
  Non-Esm  FILALCPLQE NVVDGLAVDL SRHVVYHCPC GVGRNDVLRK EETLLMVCAL LQQLRPREII FPLCFVPNAA QADRKASFAQ RLLDW 255
      Esm  FILALCPLQE NVVDGLAVDL SRHVVYHCPC GVGRNDVLRE EETLLMVCAP LQQLRPREII FPLCFIPNAA QADRKKSFAK RLVDW 255
            260                                      280                                      300                                      320                                      340
TritrypDB  IEGEGFRVEL VDVSSLSFSK GNSDGVDKIL DAHKFLAHYF QTLKLYHAVP ILSEAEPYTF HLPYASSNTT IARKHEGEVS NSSSI 340
  Non-Esm  IEGEGFRVEL VDVSSLSFSK GNSDGVDKIL DAHKFLAHYF QTLKLYHAVP ILSEAEPYTF HLPYASSNTT IARKHEGEVS NSSSI 340
      Esm  IEGEGFRVEL VDVSSLSVSK GNSDGVDKIL DAHKFLAHYF QTLKLYHAVP ILSDAEPYTF HLPYASSNTA IARKHEGGVL NSSSI 340
                                      360                                      380                                      400                                      420
TritrypDB  LWHERRVDRG LVLDATTVSN LELVSNLRDG GERGSLNQLF NRCCTNGGKR LMRSWILRPS ASSRVILARQ EAIRFIIEHK LDELW 425
  Non-Esm  LWHERRVDRG LVLDATTVSN LELVSNLRDG GERGSLNQLF NRCCTNGGKR LMRSWILRPS ASSRVILARQ EAIRFIIEHK LDELW 425
      Esm  LWHERRVDRG LVLDATTVSN LELVSNLRDG GERGSLNQLF NRCCTNGGKR LMRSWILRPS ASSRVILARQ EAIRFIIEHK LDELW 425
                           440                                      460                                      480                                      500
TritrypDB  GEGGEPETTL GIAAASPTLR AETPREHEPI CVVKRERPVG SKFETRFSSL VDVDFERNLS RLTDLKNNSD AQVAFVDPLV QYKKQ 510
  Non-Esm  GEGGEPETTL GIAAASPTLR AETPREHEPI CVVKRERPVG SKFETRFSSL VDVDFERNLS RLTDLKNNSD AQVAFVDPLV QYKKQ 510
      Esm  GEGGEPETTL GIAAAPPTVR AETPREDEPS CVVKRERPVG SKFETRFSSL VDVDFERNLS RLTDLKSNSD AQVAFVDPLV QYKKQ 510
                 520                                      540                                      560                                      580
TritrypDB  LQIIVTTVQA LEDMVAWSHD IQKGITSSPP LLKELWAQID AAAPAVTSIG NCFDRHAALA SGVIVPSQGA SSVYDEASGT LDAIE 595
  Non-Esm  LQIIVTTVQA LEDMVAWSHD IQKGITSSPP LLKELWAQID AAAPAVTSIG NCFDRHAALA SGVIVPSQGA SSVYDEASGT LDAIE 595
      Esm  LQIIITTVQA LEDMVAWSHD IQKGITSSPP LLKELWAQID AAAPAVTSIG NCFDRHAALA SGVIVPSQGT SSVYDEASGT LDAIE 595
      600                                      620                                      640                                      660                                      680
TritrypDB  GKLQGELRRL QEEVFDGTAI NYSVVGHEQF LVEVPISAVP KTPLRGFVER SRSGKSVKYV VASLEPLVES HKKAKKEKSN ALLLV 680
  Non-Esm  GKLQGELRRL QEEVFDGTAI NYSVVGHEQF LVEVPISAVP KTPLRGFVER SRSGKSVKYV VASLEPLVES HKKAKKEKSN ALLLV 680
      Esm  GKLQGELRRL QEEVFDGAAI NYSVVGHEQF LVEVPICAVP KTPLRGFVER SRSGKSVKYV VAALEPLVEA HKKAKKEKSN ALLLV 680
                                700                                      720                                      740                                      760
TritrypDB  LQNVASHMCH YFPLLYGATE ALSYFDCLLS LASLRKCGVT TAWPIVKDDA VGASLEAEQL THPFLNGDSV PNDISLDAAH GRILL 765
  Non-Esm  LQNVASHMCH YFPLLYGATE ALSYFDCLLS LASLRKCGVT TAWPIVKDDA VGASLEAREL THPFLNRDSV PNDISLDAAH GRILL 765
      Esm  LQNVASHMCH YFPLLYGATE ALSYFDCLLS LASLRKCGVT TAWPIVKDDA VGASLEAEQL THPFLNGDSV PNDISLDAAH GRILL 765
                     780                                      800                                      820                                      840
TritrypDB  LTGPNMAGKS TLMRTVAVNV IIAQMGGPIF GAFMRLATVS RIFTRIGARD ASHKGQSTLY VELSETADIL RHADPWSLCL VDELG 850
  Non-Esm  LTGPNMAGKS TLMRTVAVNV IIAQMGGPIF GAFMRLATVS RIFTRIGARD ASHKGQSTLY VELSETADIL RHADPWSLCL VDELG 850
      Esm  LTGPNMAGKS TLMRTVAVNV IIAQMGGPIF GAFMRLATVS RIFTRIGARD ASHKGQSTLY VELSETADIL RHADPWSLCL VDELG 850
          860                                      880                                      900                                      920
TritrypDB  RGTSTHDGYA IAHATLHSLK ERLPVSPLLL FSTHYHALAQ EQLGGDAVNV SSLARDHGSV VQLGYMDFAI SDTKKDGVSA ITFLY 935
  Non-Esm  RGTSTHDGYA IAHATLHSLK ERLPVSPLLL FSTHYHALAQ EQLGGDAVNV SSLAPDHGSV VQLGYMDFAI SDTKKDGVSA ITFLY 935
      Esm  RGTSTHDGYA IAHATLHSLK ERLPVSPLLL FSTHYHALAQ EQLGGDAVNV SSLARDHGSV VQLGYMDFAI SDTKKDGVSA ITFLY 935
      940                                      960                                      980                                      1.000
TritrypDB  RLVSGVCTRS YGVEVALLAG IPSSLVHMAA IKSHELASWN DRQKDIHTIR QFLHEPNAAT LFKKKKS■ 1003
  Non-Esm  CLVSGICTRS YGVEVALLAG IPSSLVHMAA IKSHELASWN DRQKDIHTIR QFLHGPNAAT LFKKKKS■ 1003
      Esm  RLVSGVCTRS YGVEVALLAG IPSSLVHMAA IKSHELASWN DRQKDIHTIR QFLHEPNAAT LFKKKKS■ 1003
```

Supplementary Figure 2: (A) DNA sequence alignment of the *T. cruzi msh6* gene as deposited at TritrypDB database and of the sequenced clones representing Esmeraldo and Non-Esmeraldo haplotypes. In pink, non conserved bases are highlighted. (**B**) Alignment of aminoacid sequence as deposit at TritrypDB database and of the translated sequenced clones representing Esmeraldo and Non-Esmeraldo haplotypes. Non conserved aminoacids are highlighted in gray.
