# Supplementary material for: Trypanosoma brucei and Trypanosoma cruzi DNA Mismatch Repair Proteins Act Differently in the Response to DNA Damage Caused by Oxidative Stress
Source: Front Cell Infect Microbiol. 2020 Apr 16;10:154. doi: 10.3389/fcimb.2020.00154 (PMC7176904; doi:10.3389/fcimb.2020.00154)
Supplement: Supplementary file 1 [file Data_Sheet_1.zip › Figure S3.PDF]

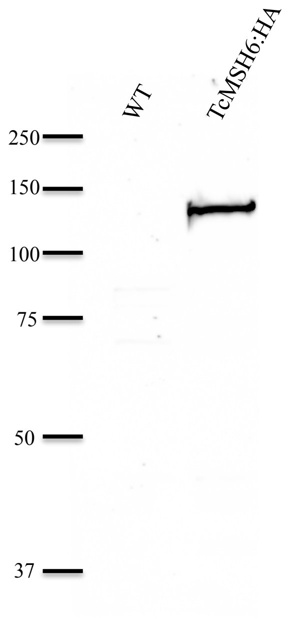

Supplementary Figure 3: Western blot of cell extract of WT and parasites expressing TcMSH6::HA. Membrane was incubated with α-HA antibody (1:2000).
