# Supplementary material for: Trypanosoma brucei and Trypanosoma cruzi DNA Mismatch Repair Proteins Act Differently in the Response to DNA Damage Caused by Oxidative Stress
Source: Front Cell Infect Microbiol. 2020 Apr 16;10:154. doi: 10.3389/fcimb.2020.00154 (PMC7176904; doi:10.3389/fcimb.2020.00154)
Supplement: Supplementary file 1 [file Data_Sheet_1.zip › Figure S5.pdf]

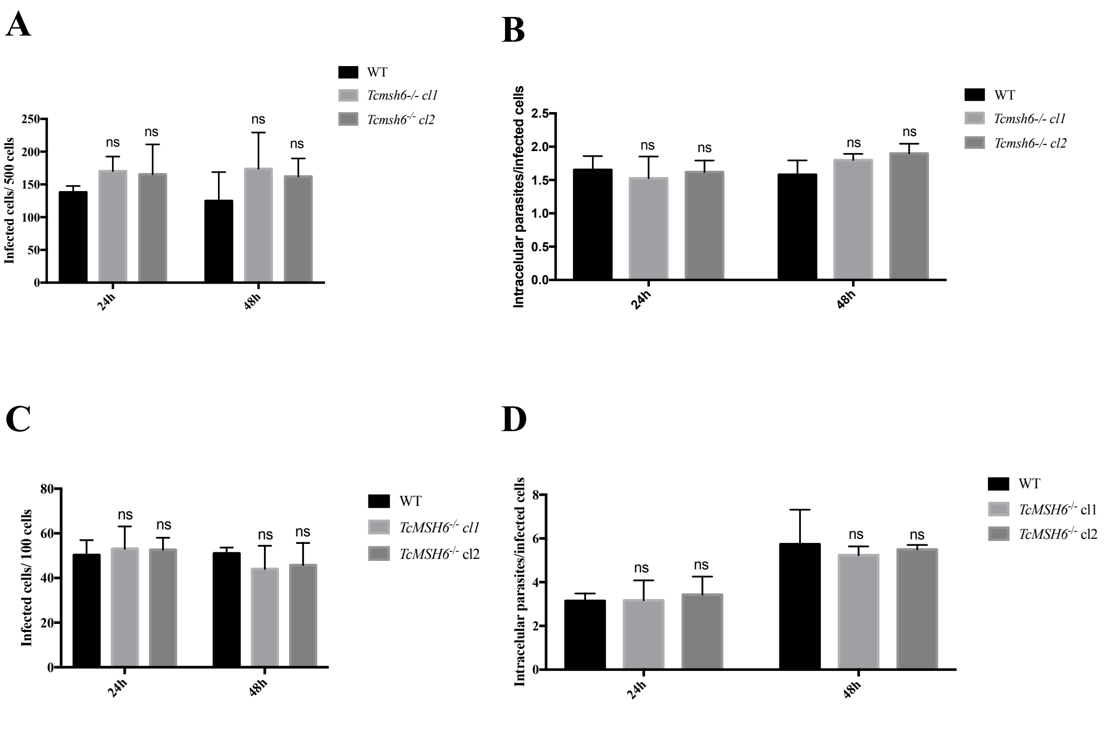


**Supplementary Figure 5: *In vitro* infectivity of *T. cruzi msh6* knockouts.** *T. cruzi* trypomastigote cells released by Vero cells infected with either WT or with two cloned cell lines of *Tcmsh6*-/- mutants were counted and equal numbers were used to infect Vero cells attached to glass coverslips. (**A**) Number of infected cells per 500 counted cells 24 and 48 hours after infections. (**B**) Number of intracellular amastigotes per infected cells. (**C**) Infection of intraperitoneal macrophages extracted from Balb/C mice with trypomastigotes released from infected Vero cells. Number of infected cells per 100 counted cells 24 and 48 hours after infections. (**D**) Number of intracellular amastigotes per infected cells. Values are expressed as means ± SD of one representative experiment performed in triplicate. Ns indicates no significant difference: two-way ANOVA with Bonferroni post-test of knockout mutants relative to wild type.
