# Supplementary material for: Trypanosoma brucei and Trypanosoma cruzi DNA Mismatch Repair Proteins Act Differently in the Response to DNA Damage Caused by Oxidative Stress
Source: Front Cell Infect Microbiol. 2020 Apr 16;10:154. doi: 10.3389/fcimb.2020.00154 (PMC7176904; doi:10.3389/fcimb.2020.00154)
Supplement: Supplementary file 2 [file Data_Sheet_2.zip › Table S1.PDF]

Table 1: Sequences of PCR primers

	Primer	Sequence
FOR KNOCKOUTS	TbMSH3-5’ (F)	5′ CATGAGCTCCTACGGGAAAATTTAGCAAGGG 3'
	TbMSH3-5’ (R)	5′ CATGCGGCCGCACGGCGGCGTTTGCTCATTTTGC 3'
	TbMSH3-3’ (F)	5′ CATTTAATTAAGAAGTCGAGTCGAATGTGTGACC 3'
	TbMSH3-3’ (R)	5′ CATCTCGAGCACACACACACTACTACTACGAGAGG 3'
	TbMSH6-5' (F)	5′ CATGAGCTCCGTGTATGTATCGGGTACACCC 3'
	TbMSH6-5' (R)	5′ CATGCGGCCGCAGGATACCAAGGGTCAGATCTCC 3'
	TbMSH6-3' (F)	5′ CATTTAATTAAGTGACGCCTTTACCAATGAGTCC 3'
	TbMSH6-3' (R)	5′ CATCTCGAGTCTTTGCTTATCCCTCCTGCCG 3'
	TcMSH6 (F)	5′ CTAGTTGACTGTGCGGACGTTTACCG 3′
	TcMSH6 (R)	5′ TGAAGGAACTGTCGTATGGTATGAAT 3′
FOR CRISPR	sgSaMSH65'	5′GGAGGCCGGAGAATTGTAATACGACTCACTATAGGAGAACAAATGAAGGAGGAAAGTTTAGTACTCTGGAAACAGAATC 3′
	sgSaMSH63'	5′GGAGGCCGGAGAATTGTAATACGACTCACTATAGGGGATACATGGACTTTGCTATAGTTTTAGTACTCTGGAAACAGAATC 3′
FOR CHECKING KNOCKOUTS	P1F	5′ TGGTACCGGGAATATGCCTCAGTGTGGA 3′
	P2R	5′ ATGCTCGAGAGAAGGCCGTAAAATC 3′
	P3R	5′ TCAGAAGAACTCGTCAAGAAGGCG 3′
	P4R	5′ TCAGAAGAACTCGTCCGGCCACA 3′
FOR TAGGING	TcMSH6HAFor	5′ ACGTGCTAGCCCTACGGGAATGGATGAC 3′
	TcMSH6HARev	5′ AAGGCTAGCCGACTTCTTTTTCTTG 3′
	TcMSH2F	5′ CGACACTAGTATGACTGATGAACG 3′
	TcMSH2R	5′ GAAGACTAGTGTAAAGCAAATAC 3′
	mycEcoRVR	5′ ATCCGATATCTCACCTAGGCAG 3′
	HA_XhoIRev	5′ CGTCTCGAGCATTACCTACTGCTAATG 3′
