# Supplementary material for: Trypanosoma brucei and Trypanosoma cruzi DNA Mismatch Repair Proteins Act Differently in the Response to DNA Damage Caused by Oxidative Stress
Source: Front Cell Infect Microbiol. 2020 Apr 16;10:154. doi: 10.3389/fcimb.2020.00154 (PMC7176904; doi:10.3389/fcimb.2020.00154)
Supplement: Supplementary file 2 [file Data_Sheet_2.zip › Table S2.PDF]

Table 2: Mass spectrometry data for *T. brucei* co-IP samples

Sample number	Protein identified	Peptide sequence	Mascot score	Mass (Da)	Matches
1	**Tb10.26.1030**<br>MSH2 DNA mismatch repair protein	R.ANITLQER.G<br>R.SIEDINQR.L<br>R.DALITQVLR.R<br>R.VGYAALNTTLR.T<br>R.ELESGEGDGDSR.E<br>K.AVELEDFGGDETK.N<br>R.FVSGQLSSLSEQYK.G<br>R.AQALFSTATPEVVQR.V<br>K.NWSGVDAVAVNDSITR.E<br>R.TLINATVDLSDENTVR.I	1541	106134	63 (63)
2	**Tb09.160.3760**<br>MSH3 mismatch repair protein	K.FGDLEATLGK.L<br>R.ATTEELSVATK.L<br>R.LAIAANEAWLAK.Q<br>R.VAFADQESASIR.S<br>K.HGAWILTGPNMGGK.S	72	103468	10 (0)
3	**Tb10.26.1030**<br>MSH2 DNA mismatch repair protein	R.FVSGQLSSLSEQYK.G<br>R.AQALFSTATPEVVQR.V	137	106134	5 (5)
4	**Tb09.160.3760**<br>MSH3 mismatch repair protein	K.FGDLEATLGK.L<br>R.ATTEELSVATK.L<br>R.LAIAANEAWLAK.Q<br>R.VAFADQESASIR.S<br>R.MGSSDSLLEGSSTFLK.E	357	103468	18 (18)
