# Supplementary material for: Trypanosoma brucei and Trypanosoma cruzi DNA Mismatch Repair Proteins Act Differently in the Response to DNA Damage Caused by Oxidative Stress
Source: Front Cell Infect Microbiol. 2020 Apr 16;10:154. doi: 10.3389/fcimb.2020.00154 (PMC7176904; doi:10.3389/fcimb.2020.00154)
Supplement: Supplementary file 2 [file Data_Sheet_2.zip › Table S3.PDF]

Table 3: Phenotypes observed for different *T. brucei* and *T. cruzi* MMR knockouts

Cell line	MNNG	H_2O_2	Cell Infection		References
			Vero	Macrophage	
T. brucei msh2-/- BSF	tolerant	susceptible	N/A	N/A	Bell, et al 2003; Machado-Silva et al, 2008
T. brucei msh2-/- PCF	tolerant	tolerant	N/A	N/A	Grazielle-Silva et al, 2015
T. brucei mlh1-/- BSF	tolerant	no difference	N/A	N/A	Bell, et al 2003; Machado-Silva et al, 2008
T. brucei mlh1-/- PCF	tolerant	no difference	N/A	N/A	Grazielle-Silva et al, 2015
T. brucei msh3-/- BSF	no difference	no difference	N/A	N/A	
T. brucei msh6-/- BSF	tolerant	no difference	N/A	N/A	
T. cruzi msh2+/-	tolerant	susceptible	not tested	not tested	Campos, et al 2011; Grazielle-Silva et al, 2015
T. cruzi msh2-/-	tolerant	tolerant	no difference	increase	Grazielle-Silva et al, 2015
T. cruzi msh6-/-	tolerant	susceptible	no difference	no difference	
